# Taxonomic revision of *Chloromonas nivalis* (Volvocales, Chlorophyceae) strains, with the new description of two snow-inhabiting *Chloromonas* species

**DOI:** 10.1371/journal.pone.0193603

**Published:** 2018-03-23

**Authors:** Ryo Matsuzaki, Hisayoshi Nozaki, Masanobu Kawachi

**Affiliations:** 1 Center for Environmental Biology and Ecosystem Studies, National Institute for Environmental Studies, Onogawa, Tsukuba, Ibaraki, Japan; 2 Department of Biological Sciences, Graduate School of Science, University of Tokyo, Hongo, Bunkyo-ku, Tokyo, Japan; Donald Danforth Plant Science Center, UNITED STATES

## Abstract

*Chloromonas nivalis* (Volvocales, Chlorophyceae) is considered a cosmopolitan species of a snow-inhabiting microalga because cysts morphologically identifiable as zygotes of the species are distributed worldwide. However, recent molecular data demonstrated that field-collected cysts identified as the zygotes consist of multiple species. Recently, we demonstrated that species identification of snow-inhabiting *Chloromonas* species is possible based on light and electron microscopy of asexual life cycles in strains and molecular phylogenetic analyses. Vegetative cells without eyespots and of inverted-teardrop shape have been reported once in North American material of *C*. *nivalis*; however, strains with such vegetative cells in snow-inhabiting species of *Chloromonas* have not been examined taxonomically in detail. Here, we used light and transmission electron microscopy together with molecular analyses of multiple DNA sequences to examine several *C*. *nivalis* strains. The morphological data demonstrated that one North American strain could be identified as *C*. *nivalis*, whereas three other strains should be re-classified as *C*. *hoshawii* sp. nov. and *C*. *remiasii* sp. nov. based on vegetative cell morphology, the number of zoospores within the parental cell wall during asexual reproduction, and whether cell aggregates (resulting from repeated divisions of daughter cells retained within a parental cell wall) were observed in the culture. This taxonomic treatment was supported by multigene phylogeny and comparative molecular analyses that included a rapidly evolving DNA region. Our molecular phylogenetic analyses also demonstrated that the North American strain of *C*. *nivalis* was phylogenetically separated from the Austrian and Japanese specimens previously identified as *C*. *nivalis* based on zygote morphology.

## Introduction

During the snow melt season, snowfields in polar regions and snowpacks in mountainous areas are sometimes stained green, red, or other colors. These events are typically caused by blooms of cold-adapted microalgae [1–3]. In green snow, species belonging to the genus *Chloromonas* Gobi (Volvocales, Chlorophyceae) are generally dominant [2,3]. The genus contains at least 16 snow-inhabiting species [4–7] in addition to approximately 130 mesophilic morphological species [8,9], all of which are unicellular, green, and biflagellate. Taxonomic studies based on consecutive light microscopy (LM) of field-collected materials from North America revealed that several previously described snow species of nonmotile chlorococcalean algae, such as *Scotiella* Fritsch spp., are actually identical to the zygotes of snow-inhabiting *Chloromonas* species [10–13].

Among the snow-inhabiting *Chloromonas* species, *C*. *nivalis* (Chodat) Hoham et Mullet was considered cosmopolitan because of the world-wide distribution of cysts morphologically identified as zygotes of this species [formerly classified as *Scotiella nivalis* (Chodat) F.E. Fritsch] based on studies of North American material [1,11]. The species was generally identified solely on the basis of zygote morphology [1,14–17] according to the species concept of previous studies [11,18]. This reliance on morphology possibly arises from the difficulty of inducing vegetative cell production from field-collected zygotes of snow-inhabiting *Chloromonas*. Very recently, *Scotiella tatrae* Kol was transferred to *C*. *nivalis* and reduced to a subspecies as *C*. *nivalis* subsp. *tatrae* Procházková et al., based on morphological and molecular data obtained from field-collected materials from Austria and Slovakia [19,20]. Recent molecular data also demonstrated that field-collected cysts morphologically identified as *C*. *nivalis* zygotes are composed of at least four distinct lineages or species, one of which is considered conspecific with the strains of *C*. *miwae* (Fukushima) Muramoto et al. [6]. In addition, it has been demonstrated that correct species identification of snow *Chloromonas* species is possible based on light and electron microscopy of asexual life cycles of cultures, as well as by molecular phylogenetic analyses [5]. Vegetative cells without eyespots and of inverted teardrop shape have been reported once in North American material of *C*. *nivalis* [11]. Subsequently, several strains from public culture collections were designated as *C*. *nivalis* [21–23], possibly based on such vegetative cell morphology. To date, three strains designated as *C*. *nivalis* (CCCryo 005–99, UTEX SNO66 and UTEX SNO74) have been examined by molecular phylogenetic analyses or LM of asexual reproductive cell morphology [19,23–26]. However, detailed descriptions of vegetative cell morphology in these strains have not been provided. In addition, molecular phylogenetic analyses suggest that these strains were not monophyletic [6,19].

Therefore, in the present study, we carried out taxonomic re-examination of strains designated as *C*. *nivalis* using detailed morphological and molecular analyses. The data demonstrate that one North American strain not previously studied could be identified as *C*. *nivalis*, whereas the other strains should be re-classified as *C*. *hoshawii* Matsuzaki et al. sp. nov. and *C*. *remiasii* Matsuzaki et al. sp. nov. We also document phylogenetic relationships between the North American strain of *C*. *nivalis* and previously examined specimens of *C*. *nivalis* zygotes.

## Materials and methods

### Cultures

Three strains assigned to *C*. *nivalis* in previous studies (CCCryo 005–99, UTEX SNO66 and UTEX SNO74) [19,23–25], one North American strain labeled as *C*. *nivalis* (UTEX SNO71) [21], and *Chloromonas* sp. strain CCCryo 047–99 (phylogenetically close to the strain CCCryo 005–99 [19]) were provided by the Culture Collection of Cryophilic Algae (CCCryo) at the Fraunhofer Institute for Cell Therapy and Immunology [22] and the Culture Collection of Algae at the University of Texas at Austin (UTEX) [21,27] (S1 Table). The cultures were maintained on AF-6 medium [28] (liquid or 1.5% agar slants) at 5°C on a 14:10-h light:dark cycle under cool-white light-emitting diodes (color temperature = 5000 K) at 35–90 μmol photons m^−2^·s^−1^.

The strain UTEX SNO74 was excluded from further analyses because light microscopic and molecular data demonstrated that it has been replaced with a species of *Trebouxia* (Trebouxiales, Trebouxiophyceae) (S1 Text; S2 Table; S1 Fig).

### Morphological observations

Light and epifluorescence microscopy were performed using a BX51 microscope (Olympus Corp., Tokyo, Japan) equipped with Nomarski differential interference optics. Transmission electron microscopy (TEM) was performed as described previously [5] using a JEM-2010 transmission electron microscope (JEOL, Tokyo, Japan). Cells in actively growing 5- to 12-day-old cultures were investigated. In addition, we carried out LM of cultures at 1, 2 and 3 months after inoculation to detect the production of cell aggregates resulting from repeated divisions of daughter cells retained within the parental cell wall [5,29].

### Molecular analysis

For molecular analysis, we used nucleotide sequences of nuclear-encoded 18S and 26S ribosomal DNA (rDNA), chloroplast-encoded ATP synthase beta subunit (*atp*B), P700 chlorophyll *a* apoprotein A2 (*psa*B) and the large subunit of RuBisCO (*rbc*L) genes, and internal transcribed spacer 2 (ITS2) region of nuclear rDNA. Sequences from five snow-inhabiting strains and of the 12 mesophilic ones (S3 Table) were determined as described previously [6,30] using newly designed specific primers (S4 Table).

For multigene phylogeny, we used four strains examined in this study (CCCryo 005–99, CCCryo 047–99, UTEX SNO66 and UTEX SNO71) as well as 28 operational taxonomic units examined in previous studies [6,9] (S3 Table). All belong to the genus *Chloromonas* sensu Pröschold et al. [31] or the *Chloromonadinia* clade [32]. The mesophilic strains (S3 Table) were treated as the outgroup according to previous results [9,24,25]. The 18S and 26S rDNA, *atp*B and *psa*B gene sequences were aligned as described previously [5,33,34]. In addition, only the first and second codon positions of the nucleotides in the *atp*B and *psa*B were used for phylogenetic analyses. This was because the third nucleotide positions of the codons had an unusual base composition and markedly higher substitution rates than the 18S and 26S rDNA and the first and second codon positions of the *atp*B and *psa*B genes [6,33,35–37]. The combined 5,497-bp data matrix of the regions was subjected to Bayesian inference (BI), maximum likelihood (ML), maximum parsimony (MP), and neighbor-joining (NJ) analyses as described in previous studies [9,33] except that IQ-TREE v. 1.4.3 [38] was used in ML analysis instead of PAUP 4.0b10 [39]. In each analysis, identical sequences were reduced to a single operational taxonomic unit. Since *rbc*L gene substitutions in *Chloromonas* are unusual and may result in artifacts [33,40], we did not concatenate the *rbc*L gene sequences with the data matrix.

For comparison of the previously published sequence data from field-collected cysts identified as *C*. *nivalis* zygotes, we performed single-gene phylogenetic analyses using 18S rDNA or *rbc*L gene sequences as described above. In addition, we set three partitions (first, second, and third codon positions) for BI and ML analysis of *rbc*L gene sequences according to a previous study [4]. Additional operational taxonomic units were selected from previous studies [19,20,41] and shown in S3 Table.

Substitution models for each phylogenetic analysis are described in S5 Table. The data matrices used in the present study are available from TreeBASE [42] (matrix accession number S22105).

Methods for annotation and prediction of secondary structures of nuclear rDNA ITS2 region were described in a previous study [5]. For detecting compensatory base changes (CBCs), the ITS2 sequences were aligned on the basis of sequence-structure analysis [43] using 4SALE [44,45].

### Nomenclature

The electronic version of this article in Portable Document Format (PDF) in a work with an ISSN or ISBN will represent a published work according to the International Code of Nomenclature for algae, fungi, and plants (Melbourne Code) (http://iapt-taxon.org/nomen/main.php), and hence the new names contained in the electronic publication of a PLOS article are effectively published under that Code from the electronic edition alone, so there is no longer any need to provide printed copies.

## Results

### Morphological observation

**Fig 1:**
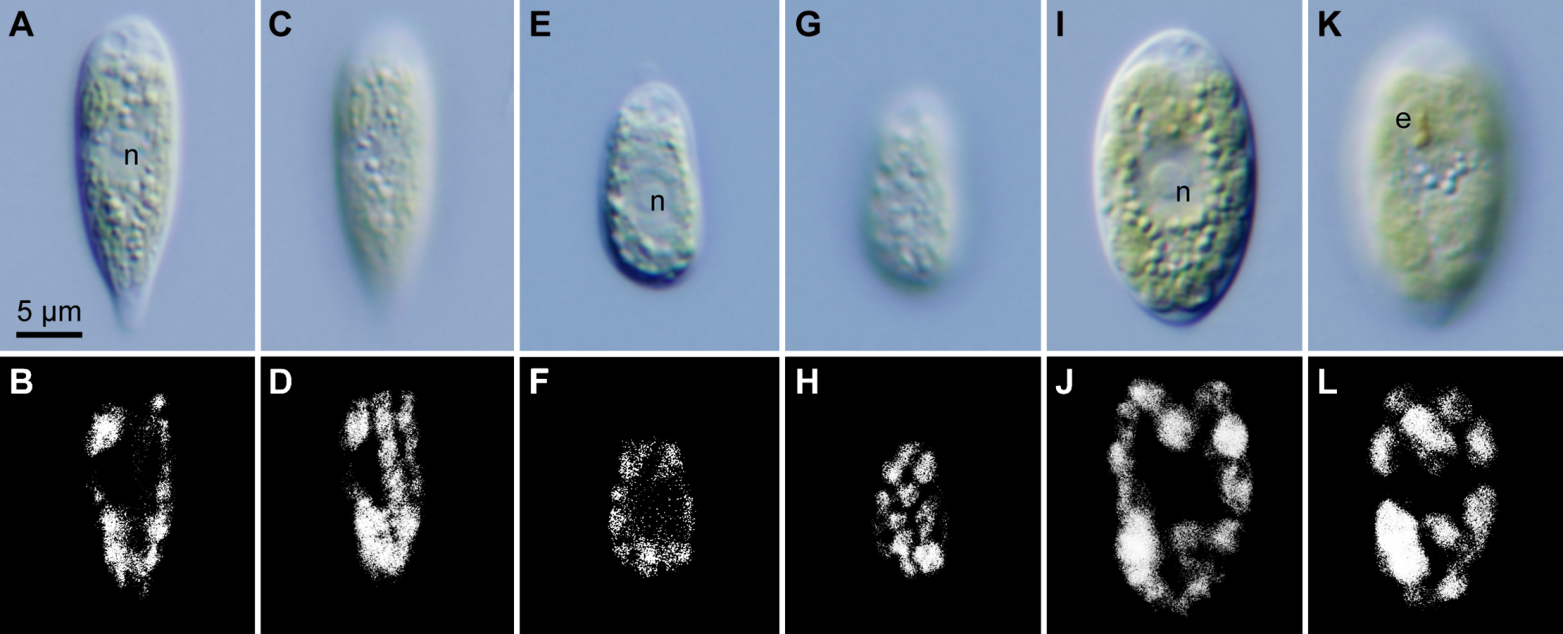
Vegetative cells of the three snow-inhabiting *Chloromonas* species: Light micrographs. Identical magnification throughout. Abbreviations: e, eyespot; n, nucleus. (A-D) *C*. *nivalis* (Chodat) Hoham et Mullet strain UTEX SNO71. (A) Optical section. (B) Epifluorescence image of (A). (C) Surface view. (D) Epifluorescence image of (C). (E-H) *C*. *hoshawii* Matsuzaki et al. sp. nov. strain UTEX SNO66. (E) Optical section. (F) Epifluorescence image of (E). (G) Surface view. (H) Epifluorescence image of (G). (I-L) *C*. *remiasii* Matsuzaki et al. sp. nov. strain CCCryo 005–99. (I) Optical section. (J) Epifluorescence image of (I). (K) Surface view. (L) Epifluorescence image of (K).

**Fig 2:**
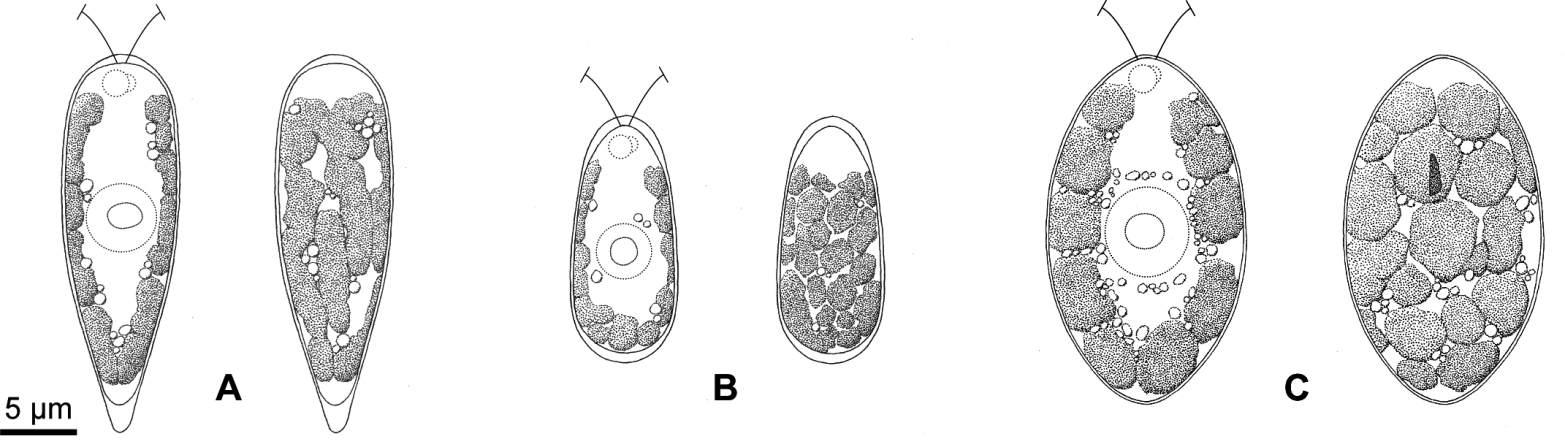
Vegetative cells of the three snow-inhabiting *Chloromonas* species: Line drawings. Identical magnification throughout. Left, optical section. Right, surface view. (A) *C*. *nivalis* (Chodat) Hoham et Mullet. (B) *C*. *hoshawii* Matsuzaki et al. sp. nov. (C) *C*. *remiasii* Matsuzaki et al. sp. nov.

Light and epifluorescence microscopy (Figs 1 and 2) demonstrated that the strains could be subdivided into three morphological species (*C*. *hoshawii*, *C*. *nivalis*, and *C*. *remiasii*) based on differences in cell shape and size, chloroplast morphology, presence of eyespots, number of zoospores formed within the parental cell wall during asexual reproduction, and presence of cell aggregates (aggregates of 16 or more cells resulting from repeated divisions of daughter cells retained within a parental cell wall [5,29]) in culture (Table 1). In *C*. *nivalis* strain UTEX SNO71, vegetative cells had an inverted-teardrop shape with a prominent posterior tail (Figs 1A and 2A). On the other hand, vegetative cells of *C*. *hoshawii* strain UTEX SNO66 were ellipsoidal to elongate-ovoid (Figs 1E and 2B), and those of *C*. *remiasii* strains CCCryo 005–99 and CCCryo 047–99 were ellipsoidal to spindle-shaped (Figs 1I and 2C); a prominent posterior tail was not observed in the cells of the latter two species in culture. The three species lacked a prominent anterior papilla (Figs 1A, 1E, 1I and 2A–2C). In some cells of *C*. *hoshawii*, cell wall became thicker at the anterior and posterior cell end (Fig 2B). The vegetative cell length of *C*. *hoshawii* (13.8–18.6 μm) was smaller than that of *C*. *nivalis* (20.2–28.6 μm) or of *C*. *remiasii* (18.2–30.8 μm) (Table 1). Although the chloroplasts of the three species were cup-shaped (Figs 1A, 1B, 1E, 1F, 1I, 1J and 2A–2C), the surface view of the chloroplast of *C*. *nivalis* appeared as elongate-ovoid or elongate-cylindrical platelets (Figs 1C, 1D and 2A), whereas that of *C*. *hoshawii* and *C*. *remiasii* appeared as angular discs (Figs 1G, 1H, 1K, 1L, 2B and 2C). Vegetative cells of *C*. *remiasii* possessed an ellipsoidal or elongate-D-shaped eyespot positioned in the anterior third of the cell (Figs 1K and 2C). In contrast, eyespots were not observed in the cells of *C*. *hoshawii* or of *C*. *nivalis* (Figs 1C, 1G, 2A and 2B).

**Table 1:** Morphological characteristics of the three snow-inhabiting *Chloromonas* species.

	*C*. *nivalis*	*C*. *hoshawii* sp. nov.	*C*. *remiasii* sp. nov.
Strain(s)	UTEX SNO71	UTEX SNO66	CCCryo 005–99, CCCryo 047–99
Cell shape	inverted teardrop, prominent posterior tail	ellipsoidal to elongate-ovoid, rounded posterior end	ellipsoidal to spindle, rounded posterior end
Cell width × cell length	6.6–12.4 μm × 20.2–28.6 μm	4.9–9.3 μm × 13.8–18.6 μm	10.2–15.6 μm × 18.2–30.8 μm
Chloroplast shape	cup-shaped, seemingly composed of elongate platelets	cup-shaped, seemingly composed of angular discs	cup-shaped, seemingly composed of angular discs
Eyespot	absent	absent	present
Number of zoospores formed within the parental cell wall	up to 16	2 or 4 (rarely 8)	2 or 4 (rarely 8)
Cell aggregates in culture	not observed	not observed	observed

Asexual reproduction of the three species (S2 Fig) occurred through zoospore formation by successive cell divisions, as described in a report of *C*. *nivalis* from North America [11]. Immediately prior to the first cell division, the parental contractile vacuoles moved to the equator of the cell by protoplast rotation (arrows in S2A, S2C and S2E Fig). Typically, up to four zoospores were seen in *C*. *hoshawii* (S2D Fig) and *C*. *remiasii* (S2F Fig), and up to 16 in *C*. *nivalis* (S2B Fig) (Table 1). In addition, cell aggregates resulting from repeated divisions of daughter cells retained within the parental cell wall [5,29] were produced in fresh (5- to 12-day-old) cultures as well as in old (almost or more than one-month-old) cultures of *C*. *remiasii* (S3 Fig). In contrast, such cell aggregates were not observed in the other two species (Table 1). Sexual reproduction or hypnospore formation was not observed in the three species. All three species failed to grow at 20°C after two weeks of cultivation, as described in previous reports of other species of snow-inhabiting *Chloromonas* [5,6,46].

**Fig 3:**
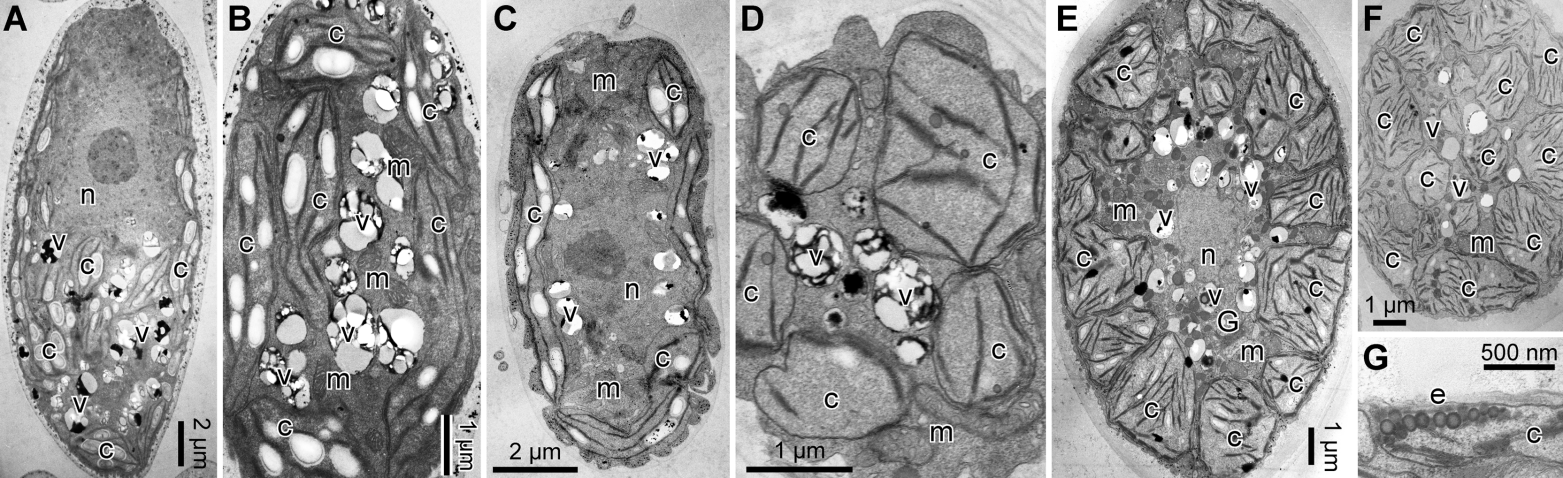
Vegetative cells of the three snow-inhabiting *Chloromonas* species: Transmission electron micrographs. Abbreviations: c, chloroplast; e, eyespot; G, Golgi body; m, mitochondrion; n, nucleus; v, vacuole with crystalline content. (A, B) *C*. *nivalis* (Chodat) Hoham et Mullet strain UTEX SNO71. (A) Longitudinal cell section. (B) Tangential cell section. (C, D) *C*. *hoshawii* Matsuzaki et al. sp. nov. strain UTEX SNO66. (C) Longitudinal cell section. (D) Tangential cell section. (E-G) *C*. *remiasii* Matsuzaki et al. sp. nov. strain CCCryo 005–99. (E) Longitudinal cell section. (F) Tangential cell section. (G) Eyespot composed of a single layer of electron-dense globules.

TEM (Fig 3) showed that each cell of the three species possessed a nucleus, and a cup-shaped chloroplast without pyrenoid matrices (Fig 3A, 3C and 3E). As in other snow-inhabiting *Chloromonas* species [5], mitochondria and Golgi bodies were present mainly between the nucleus and chloroplast. Several small vacuoles with crystalline content were observed in the cytoplasm of the three species (Fig 3A–3F). Tangential sections of *C*. *nivalis* showed the chloroplast profiles to be almost elongate in shape (Fig 3B). In contrast, chloroplasts of *C*. *hoshawii* and *C*. *remiasii* were generally angular in shape (Fig 3D and 3F). LM surface views of the chloroplasts correlated with TEM images; the chloroplasts appeared to be composed of elongate platelets or angular discs (Figs 1C, 1D, 1G, 1H, 1K, 1L and 2A–2C). The eyespot of *C*. *remiasii* was comprised of a single layer of electron-dense globules (Fig 3G). Such structures were not seen in *C*. *hoshawii* or in *C*. *nivalis*, even under TEM.

### Molecular phylogenetic analyses

**Fig 4:**
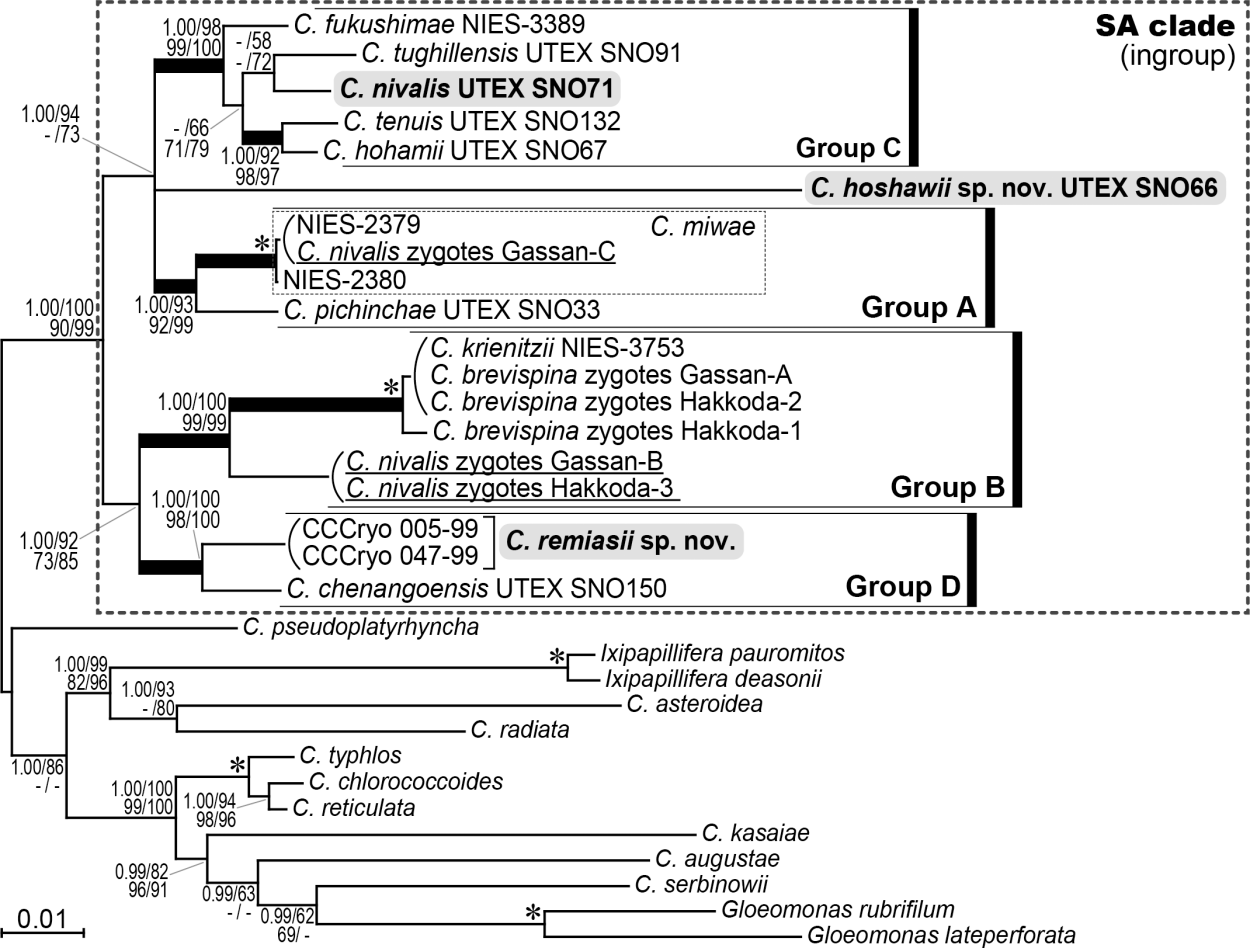
Bayesian phylogenetic tree of snow-inhabiting *Chloromonas* spp. based on 5,497 base pairs from 18S and 26S rDNA, and the first and the second codon positions of *atp*B and *psa*B. *C*. *nivalis* zygote specimens (field-collected samples) are underlined. Corresponding posterior probabilities (PP; 0.95 or more) are shown at top left. Numbers shown in top right, bottom left and bottom right indicate bootstrap values (BV; 50% or more) in maximum likelihood (ML), maximum parsimony (MP) and neighbor-joining (NJ) analyses, respectively. Branches within the SA clade (recovered at 1.00 PP and 90% or more BV in ML, MP and NJ analyses) are shown by thick lines. Asterisk indicates 1.00 PP in BI and 100% BV in ML, MP and NJ analyses.

Phylogenetic analyses (based on the sequences of 18S and 26S rDNA, and the first and the second codon positions of *atp*B and *psa*B), revealed four robust monophyletic groups of snow-inhabiting *Chloromonas* species (A–D) resolved with 1.00 posterior probabilities (PP) in BI and 92–100% bootstrap values (BV) in ML, MP and NJ analyses (Fig 4). *Chloromonas nivalis* strain UTEX SNO71 and both *C*. *remiasii* strains (CCCryo 005–99 and CCCryo 047–99) were included within groups C and D, respectively, whereas *C*. *hoshawii* strain UTEX SNO66 was positioned outside of the four groups and therefore represents an independent lineage. Group C contained *C*. *fukushimae* Matsuzaki et Nozaki, *C*. *hohamii* H.U. Ling et Seppelt, *C*. *nivalis*, *C*. *tenuis* Matsuzaki et Nozaki, and *C*. *tughillensis* Hoham et al. Within the group, *C*. *nivalis* was sister to *C*. *tughillensis* with 58% and 72% BV in ML and NJ analyses, respectively. *Chloromonas hohamii* and *C*. *tenuis* formed another clade supported by 1.00 PP in BI and 92–98% BV in ML, MP and NJ analyses. The two subclades were sister to each other (66–79% BV in ML, MP and NJ analyses), and *C*. *fukushimae* was the most basally located strain. Group D was composed of *C*. *chenangoensis* Hoham et al. and *C*. *remiasii*. Importantly, Japanese specimens identified as *C*. *nivalis* zygotes (Gassan-B, Gassan-C and Hakkoda-3 [6]) were positioned within groups A and B, and were well separated from the North American *C*. *nivalis* strain UTEX SNO71. Group A comprised *C*. *pichinchae* Wille strain UTEX SNO33 together with a small robust clade containing the two strains of *C*. *miwae* and a specimen of *C*. *nivalis* zygotes, Gassan-C; this subclade was considered a single species in a recent molecular analysis [6]. Group B contained two *C*. *nivalis* zygote specimens (Gassan-B and Hakkoda-3), three *C*. *brevispina* (F.E. Fritsch) Hoham et al. zygote specimens (Gassan-A, Hakkoda-1 and Hakkoda-2 [6]) and *C*. *krienitzii* Matsuzaki et Nozaki strain NIES-3753. In the present multigene phylogenetic tree, the four robust monophyletic groups and one independent lineage of *C*. *hoshawii* were subdivided into two large clades: one composed of groups A and C together with *C*. *hoshawii* (1.00 PP in BI and 94% and 73% BV in ML and NJ analyses, respectively), and the other constructed of groups B and D (1.00 PP in BI and 73–92% BV in ML, MP and NJ analyses). Within the former clade, phylogenetic relationships among groups A and C and *C*. *hoshawii* were not resolved.

Further comparison of phylogenetic relationships between *C*. *nivalis* strain UTEX SNO71 and field-collected *C*. *nivalis* zygote specimens examined in previous studies [19,20,41] was performed by single-gene phylogenetic analyses using 18S rDNA and *rbc*L sequences (S4 and S5 Figs). Both trees reconstructed the monophyletic groups B–D which were robustly resolved in the multigene phylogenetic tree (Fig 4), but statistical support values for monophyly were lower. Group A in Fig 4 was recovered only in the *rbc*L-based tree (S5 Fig). In the 18S rDNA- and *rbc*L-based trees (S4 and S5 Figs), the Austrian *C*. *nivalis* zygote specimen (P24/DR4 [19,20]) and the Slovak *C*. *nivalis* subsp. *tatrae* zygote specimen (LP01 [20]) were positioned within group B and formed a small robust clade (1.00 PP in BI and >89% BV in ML, MP and NJ analyses). This subclade was sister to the Japanese *C*. *nivalis* zygote specimens (Gassan-B and Hakkoda-3 [6]) with 1.00 PP in BI and 74–83% and 83–94% BV in ML, MP and NJ analyses in 18S rDNA- and *rbc*L-based tree, respectively. In addition, the two Japanese *C*. *nivalis* zygote specimens (Gassan-NIV1 and Gassan-NIV2 [41]) were positioned outside of groups A–D in the phylogenetic tree of *rbc*L sequences (S5 Fig). However, *C*. *nivalis* strain UTEX SNO71 was included within group C in 18S rDNA- and *rbc*L-based trees, and was phylogenetically separated from the Austrian, Japanese and Slovak *C*. *nivalis* zygote specimens.

### Comparative molecular analyses

**Fig 5:**
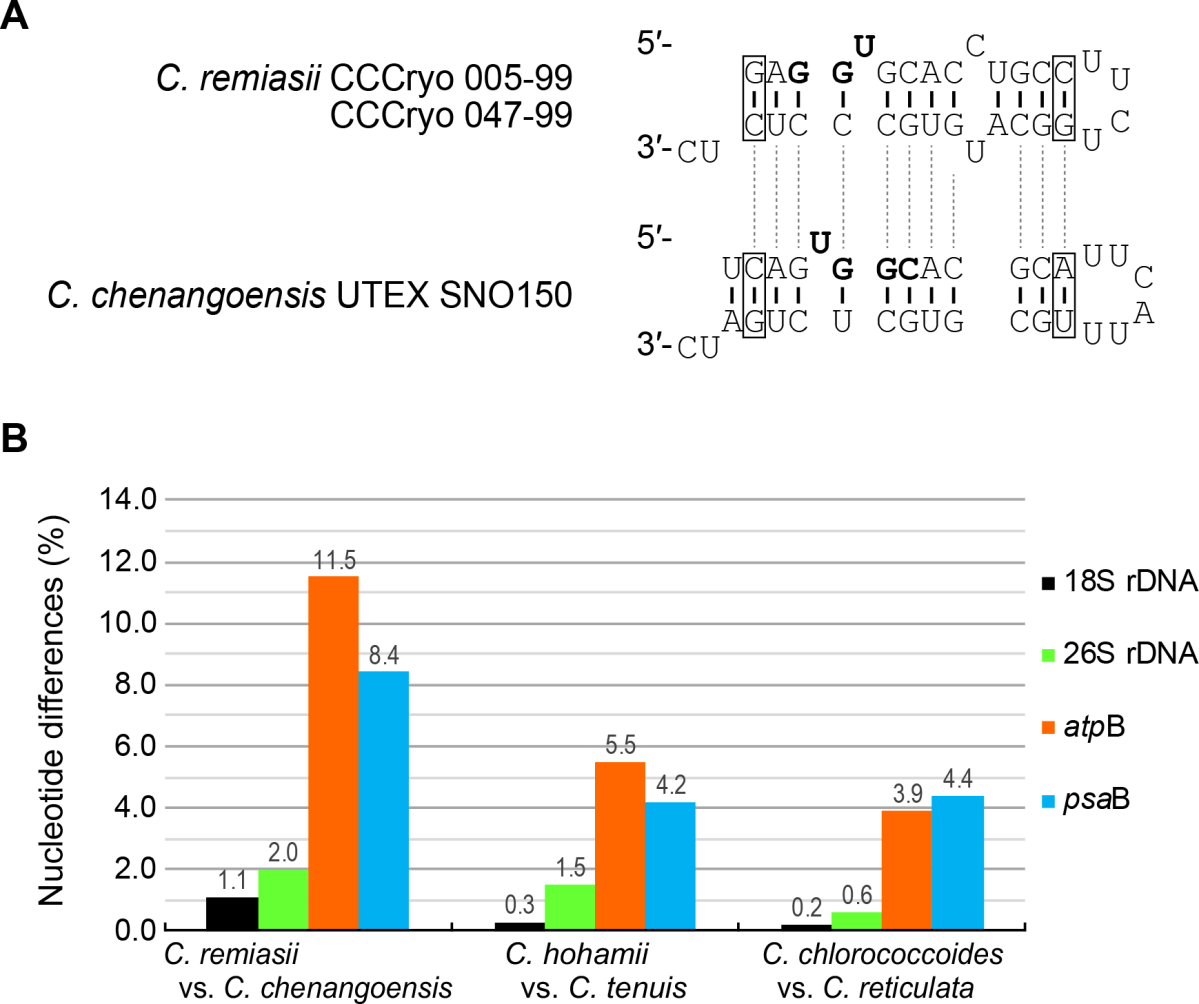
Genetic differences between *Chloromonas remiasii* Matsuzaki et al. sp. nov. and *C*. *chenangoensis* Hoham et al. (A) Comparison of the most conserved region (near the apex of helix III encompassing the YGGY motif) of nuclear rDNA ITS2 secondary structures. Open box indicates compensatory base change. Boldface marks the YGGY motif. For the complete nuclear rDNA ITS2 secondary structures, see S6 and S7 Figs. (B) Nucleotide differences (%) from pairwise comparisons in four genes. Black: nuclear-encoded 1,748 bases of 18S ribosomal DNA (rDNA). Green: nuclear-encoded 2,020 bases of 26S rDNA. Red: chloroplast-encoded 1,128 bases of ATP synthase beta subunit gene (*atp*B). Blue: chloroplast-encoded 1,392 bases of P700 chlorophyll *a* apoprotein A2 gene (*psa*B). Note that the sequences from *Chloromonas remiasii* strains CCCryo 005–99 and CCCryo 047–99 were identical. The nucleotide differences between snow-inhabiting and mesophilic sister species [*C*. *hohamii* H.U. Ling et Seppelt vs. *C*. *tenuis* Matsuzaki et Nozaki; and *C*. *chlorococcoides* (H. Ettl et K. Schwarz) Matsuzaki et al. vs. *C*. *reticulata* (Goroschankin) Gobi] are according to the previous study [5].

To verify separation of *C*. *remiasii* and *C*. *chenangoensis*, which were sister to each other (Fig 4), we compared the secondary structures of the nuclear rDNA ITS2 region. The predicted secondary structures (S6 and S7 Figs) possessed four helices, a U-U mismatch in helix II (S6 and S7 Figs, arrowheads), and the YGGY motif on the 5′ side near the apex of helix III (S6 Fig and S7 Fig, boldface). All these features are common structural hallmarks of eukaryote nuclear rDNA ITS2 secondary structures [47–50]. In *C*. *remiasii* and *C*. *chenangoensis*, at least two CBCs were detected near the apex of helix III encompassing the YGGY motif (the most conserved region of nuclear rDNA ITS2 secondary structures [48,49]) (Fig 5A). In addition, we estimated the uncorrected p-distances in nuclear-encoded 18S and 26S rDNA, and in chloroplast-encoded *atp*B and *psa*B genes, for *C*. *remiasii* and *C*. *chenangoensis*. The nucleotide differences between the two species were much larger than those between snow-inhabiting *C*. *hohamii* and *C*. *tenuis*, and also between mesophilic *C*. *chlorococcoides* (H. Ettl et K. Schwarz) Matsuzaki et al. and *C*. *reticulata* (Goroschankin) Gobi, each pair being sister species previously delineated by morphological and molecular data [5,51] (Fig 5B).

## Discussion

Zygotes or cysts morphologically identified as *C*. *nivalis* have been reported from various localities of the world [1,11,18]. However, motile vegetative cells directly obtained from such dormant cells have never been reported [11,19]; the partial life cycle (from vegetative cells to zygotes) of *C*. *nivalis* was observed only in North American field-collected material [11]. The type locality of *Pteromonas nivalis* Chodat (the basionym of *C*. *nivalis*) is in the French Alps [52]; however, the original species description lacks information on motile vegetative cells, and neither a strain nor sequences are available. Recent robust molecular data indicated that Japanese field-collected cysts morphologically identical to the North American *C*. *nivalis* zygotes (= *P*. *nivalis* and *S*. *nivalis* [11]) contain multiple species [6]. Thus, *C*. *nivalis* should be circumscribed by the vegetative morphology reported from the North American material [11].

The light microscopic features of the North American strain UTEX SNO71, which has not been examined in previous studies, were consistent with those of North American *C*. *nivalis* [11] with respect to cell shape, chloroplast morphology, and the number of zoospores formed within the parental cell wall (Table 1; S2 Text). Thus, we consider the strain UTEX SNO71 as *C*. *nivalis*, although zygotes were not observed in our study. Contrary, the vegetative morphology of strains previously designated as *C*. *nivalis* (CCCryo 005–99 and UTEX SNO66) [19,23,24] differed from that of strain UTEX SNO71 (Table 1). In addition, these three strains were phylogenetically well separated from each other (Fig 4). Therefore, based on morphology and phylogeny of vegetative cells, we re-classified strains CCCryo 005–99 and UTEX SNO66 as *C*. *remiasii* and *C*. *hoshawii*, respectively.

LM and TEM showed that chloroplasts of *C*. *hoshawii* and *C*. *remiasii* lack pyrenoids (Figs 1E, 1I and 3C–3F), and the species are robustly positioned within *Chloromonadinia* clade (Fig 4). These characteristics correspond to both traditional [8,53] and phylogenetically revised [31] concepts of the genus *Chloromonas*. Among the snow-inhabiting species of the genus, *C*. *hoshawii* resembles *C*. *chenangoensis* and *C*. *pichinchae* in having an ellipsoidal or elongate-ovoid vegetative cell with rounded anterior and posterior ends, and a chloroplast which appeared to be composed of angular discs and had no eyespot (Figs 1E and 2B; S2 Text; S6 Table) [5,8,10,25]. However, *C*. *hoshawii* differs from *C*. *pichinchae* in that it does not produce cell aggregates in old cultures (S2 Text; S6 Table) [5]. Maximum cell width is less than 10 μm in *C*. *hoshawii*, whereas vegetative cell width of *C*. *chenangoensis* is up to 17.5 μm (S2 Text; S6 Table) [5,25]. Furthermore, the phylogenetic position of *C*. *hoshawii* strain UTEX SNO66 is separated from those of *C*. *chenangoensis* strain UTEX SNO150 and *C*. *pichinchae* strain UTEX SNO33 (Fig 4). On the other hand, *C*. *remiasii* is very similar to *C*. *alpina* Wille in possessing an ellipsoidal vegetative cell with rounded anterior and posterior ends, and a chloroplast seemingly composed of angular discs and having an eyespot (Figs 1I, 1K and 2C; S2 Text; S6 Table) [8,53,54]. However, *C*. *remiasii* differs from *C*. *alpina* (of which no sequences are available) in cell size (10.2–15.6 μm wide × 18.2–30.8 μm long vs. 4–7 μm wide × 9–12 μm long, respectively; S6 Table) [8,53,54]. Although the present phylogenetic results demonstrate that *C*. *remiasii* is sister to *C*. *chenangoensis* (Fig 4), *C*. *remiasii* can be distinguished from *C*. *chenangoensis* in having an eyespot on the chloroplast and in producing cell aggregates in culture (Figs 1K and 2C; S3 Fig; S2 Text; S6 Table) [5,25]. In addition, the two species had at least two CBCs in the most conserved region of nuclear rDNA ITS2 secondary structures (Fig 5A). The CBCs correlate with the separation of biological species, according to [49]. Furthermore, genetic differences in the four genes between these two species were much larger than those between snow-inhabiting *C*. *hohamii* and *C*. *tenuis*, or between mesophilic *C*. *chlorococcoides* and *C*. *reticulata*, each pair being sister species delineated by morphological and molecular data [5,51] (Fig 5B). Therefore, the separation of *C*. *remiasii* and *C*. *chenangoensis* was supported by morphological and molecular data, and apparently they have different patterns of geographic distribution (Arctic Svalbard vs. Arizona, USA [21,22,23,25]).

Although neither *C*. *hoshawii* nor *C*. *remiasii* could grow at 20°C, a comparison of their vegetative morphology with that of mesophilic *Chloromonas* species was performed: The mesophilic species *C*. *enteromorphae* (Brabez) Gerloff et H. Ettl, *C*. *eumaculata* P.C. Silva, *C*. *gutenbrunnensis* Wawrik, and *C*. *granulata* (L.Ş. Péterfi) Gerloff et H. Ettl resemble *C*. *hoshawii* and *C*. *remiasii* in that the cells are ovoid to ellipsoidal with rounded anterior and posterior ends, and they have a cup-shaped chloroplast seemingly composed of angular discs [8,53]. However, *C*. *hoshawii* differs from the mesophilic species by the lack of an eyespot on the chloroplast (Figs 1G and 2B) [8,53,55–58]. The eyespot of *C*. *remiasii* is positioned in the anterior third of the cell, whereas those of *C*. *eumaculata* and *C*. *gutenbrunnensis* are located near the equator or in the posterior third of the cell, respectively [8,53,55,58]. The cell wall of *C*. *granulata* is quite swollen; this trait was not observed in vegetative cells of *C*. *remiasii* (Figs 1I and 2C) [8,53,56]. The nucleus of *C*. *remiasii* is positioned in the middle of the protoplast (Figs 1I and 2C), whereas the nucleus is in the posterior third of the cell in *C*. *enteromorphae* [8,53,57]. Moreover, the vegetative cells of *C*. *remiasii* are smaller than those of *C*. *enteromorphae* (up to 30.8 μm long vs. up to 44 μm long, respectively) (Table 1) [57]. Thus, *C*. *hoshawii* and *C*. *remiasii* represent two new morphological species of the genus *Chloromonas*.

Molecular phylogenetic analyses (Fig 4; S4 and S5 Figs) demonstrated that the North American strain morphologically assignable to *C*. *nivalis* from North America is phylogenetically separated from Austrian, Japanese and Slovak field-collected zygote specimens earlier identified as *C*. *nivalis* [6,19,20,41]. Therefore, taxonomic re-examination of the latter specimens should be carried out based on their vegetative morphologies. In addition, scanning electron microscopic features of the zygotes might also help their taxonomic revision [20]. Although no one has successfully induced the production of motile vegetative cells from field-collected zygotes of snow-inhabiting *Chloromonas* under controlled laboratory conditions [10–13,19,23], our recent study provided a practical method for molecular identification of such zygotes by using data obtained from accurately identified cultures [6]. Thus, further taxonomic studies of cultured snow-inhabiting *Chloromonas* are required to reveal the correct affiliation of field-collected cysts currently identified as *C*. *nivalis* zygotes.

### Taxonomic treatments

*Chloromonas hoshawii* Matsuzaki, Nozaki et Kawachi sp. nov.

Vegetative cells solitary, having two flagella, without a prominent anterior papilla. Cells ellipsoidal or elongate-ovoid; 4.9–9.3 μm wide and 13.8–18.6 μm long. Cells with a central nucleus and a single cup-shaped chloroplast. Chloroplast seemingly composed of angular discs, showing irregular incisions on the surface, without an eyespot and pyrenoids. Asexual reproduction by formation of generally two or four zoospores, with rotation of the protoplast before the first cell division. Cell aggregates not observed in culture.

Holotype: Specimen TNS-AL-58946 deposited at TNS (National Museum of Nature and Science, Tsukuba, Japan); material consists of resin-embedded vegetative cells from strain UTEX SNO66.
Strain examined: UTEX SNO66 (Table 1).
Etymology: The species epithet *hoshawii* is in honor of Dr. Robert W. Hoshaw who contributed greatly to the taxonomy of green algae (e.g. [59,60]). He participated in collection of material from which the authentic strain of this species was isolated [21].
Type locality: Mt. Lemmon, Arizona, USA [21,24].

*Chloromonas remiasii* Matsuzaki, Nozaki et Kawachi sp. nov.

Vegetative cells solitary, having two flagella, without a prominent anterior papilla. Cells ellipsoidal or spindle-shaped; 10.2–15.6 μm wide and 18.2–30.8 μm long. Cells with a central nucleus and a single cup-shaped chloroplast. Chloroplast seemingly composed of angular discs, showing irregular incisions on the surface, with an eyespot and without pyrenoids. Eyespot ellipsoidal to elongate D-shaped, positioned in the anterior third of the cell, composed of a single layer of globules. Asexual reproduction by formation of generally two or four zoospores, with rotation of the protoplast before the first cell division. Cell aggregates observed in culture.

Holotype: Specimen TNS-AL-58947 deposited at TNS (National Museum of Nature and Science, Tsukuba, Japan); material consists of resin-embedded vegetative cells from strain CCCryo 005–99.
Strains examined: CCCryo 005–99, CCCryo 047–99 (Table 1).
Etymology: The species epithet *remiasii* is in honor of Dr. Daniel Remias, who has contributed greatly to the ecology and physiology of snow-inhabiting microalgae (e.g. [19,26,61]).
Type locality: Bjørnhamna, Reuschhalvøya, Spitsbergen, Svalbard, Norway [22,23].
Remarks: A previous study [23] suggested relationship between the strain CCCryo 005–99 and field-collected cysts or zygotes, both of which were collected at the same location in Svalbard. The cysts resemble North American *C*. *nivalis* zygotes in having spindle-shaped cell with several longitudinal, slightly helical ridges on the cell wall extended partially to the poles. Since molecular data of the cysts are not available and sexual reproduction of *C*. *remiasii* has not been observed, we could not confirm this possible relationship.

## Supporting information

S1 Fig Vegetative cell of the strain UTEX SNO74. Abbreviations: c, chloroplast; n, nucleus; p, pyrenoid. (A) Optical section focused on a pyrenoid. (B) Surface view. The strain [formerly designated as *Chloromonas nivalis* (Chodat) Hoham et Mullet] was not used in course of this study since the strain might be replaced with contamination by the species of the genus *Trebouxia* (see S1 Text; S2 Table).
(TIF)

S2 Fig Asexual reproduction of three snow-inhabiting *Chloromonas* species. All at identical magnification. Arrows in A, C, E indicate position of each contractile vacuole originating from the parent cell. (A, B) *C*. *nivalis* (Chodat) Hoham et Mullet strain UTEX SNO71. (A) Immediately prior to the first transverse division. (B) Sixteen daughter cells within the parental cell wall. Note that only 12 of the 16 cells are recognized. (C, D) *C*. *hoshawii* Matsuzaki et al. sp. nov. strain UTEX SNO66. (C) Immediately prior to the first transverse division. (D) Four daughter cells within the parental cell wall. (E, F) *C*. *remiasii* Matsuzaki et al. sp. nov. strain CCCryo 005–99. (E) Immediately prior to the first transverse division. (F) Four daughter cells within the parental cell wall.
(TIF)

S3 Fig Cell aggregates in cultures of *Chloromonas remiasii* Matsuzaki et al. sp. nov. Aggregates result from repeated divisions of daughter cells retained in parental cell walls (double arrowhead). Open arrowhead indicates a daughter cell wall surrounding offspring of a daughter cell. All at the identical magnification. (A) Strain CCCryo 005–99 after 7 days in liquid AF-6 medium. (B) Strain CCCryo 047–99 after 3 months on 1.5% agar slant of AF-6.
(TIF)

S4 Fig Bayesian phylogenetic tree of snow-inhabiting *Chloromonas* spp. based on 18S ribosomal DNA sequences. *C*. *nivalis* zygote specimens (Field-collected samples) are underlined, and the Austrian *C*. *nivalis* zygote specimen (P24/DR4 [19]) and the Slovak *C*. *nivalis* subsp. *tatrae* zygote specimen (LP01 [20]) are shadowed in black. Groups A–D are as indicated in Fig 4. The corresponding posterior probabilities (PP, 0.95 or more) are shown at the top left. Numbers shown in top right, bottom left and bottom right indicate bootstrap values (BV, 50% or more) from maximum likelihood (ML), maximum parsimony (MP) and neighbor-joining (NJ) analyses. Asterisk indicates 1.00 PP in BI and 100% BV in ML, MP, and NJ analyses.
(TIF)

S5 Fig Bayesian phylogenetic tree of snow-inhabiting *Chloromonas* spp. based on the large subunit of RuBisCO gene sequences. *C*. *nivalis* zygote specimens (Field-collected samples) are underlined, and the Austrian and Japanese *C*. *nivalis* zygote specimens examined in the previous studies (P24/DR4 [19,20], and Gassan-NIV1 and Gassan-NIV2 [41], respectively) and the Slovak *C*. *nivalis* subsp. *tatrae* zygote specimen (LP01 [20]) are shadowed in black. Groups A–D are as in Fig 4. The corresponding posterior probabilities (PP, 0.95 or more) are shown at the top left. Numbers shown in top right, bottom left and bottom right indicate bootstrap values (BV, 50% or more) from maximum likelihood (ML), maximum parsimony (MP) and neighbor-joining (NJ) analyses. Asterisk indicates 1.00 PP in BI and 100% BV in ML, MP and NJ analyses.
(TIF)

S6 Fig Secondary structure of nuclear ribosomal DNA (rDNA) internal transcribed spacer 2 (ITS2) transcript of *Chloromonas remiasii* Matsuzaki et al. sp. nov. strain CCCryo 005–99. The 3′ end of the 5.8S ribosomal RNA (rRNA) and the 5′ end of the 26S rRNA are shown (DDBJ/ENA/GenBank accession number: HQ404862). The sequence from *C*. *remiasii* strains CCCryo 005–99 is identical to that from CCCryo 047–99 (LC360496). Note U-U mismatch in helix II (arrowheads) and the YGGY motif on the 5′ side near the apex of helix III (boldface), common structural hallmarks of eukaryotic nuclear rDNA ITS2 secondary structures [47,50].
(TIF)

S7 Fig Secondary structure of nuclear ribosomal DNA (rDNA) internal transcribed spacer 2 (ITS2) transcript of *Chloromonas chenangoensis* strain UTEX SNO150. The 3′ end of the 5.8S ribosomal RNA (rRNA) and the 5′ end of the 26S rRNA are shown (DDBJ/ENA/GenBank accession number: LC360497). Note U-U mismatch in helix II (arrowheads) and the YGGY motif on the 5′ side near the apex of helix III (boldface), common structural hallmarks of eukaryotic nuclear rDNA ITS2 secondary structures [47,50].
(TIF)

S1 Table Strains examined in this study. (DOCX)

S2 Table BLASTn results using two gene sequences of the four strains as queries against nucleotide collection. (DOCX)

S3 Table Taxa/specimens/strains in the present molecular analyses (Figs 4 and 5; S4 and S5 Figs) and DDBJ/ENA/GenBank accession numbers of the five genes. (DOCX)

S4 Table Primers for amplification and sequencing of P700 chlorophyll *a* apoprotein A2 gene from *Chloromonas remiasii* strains. (DOCX)

S5 Table Substitution models applied to respective data matrices of the present phylogenetic analyses (Fig 4; S4 and S5 Figs). (DOCX)

S6 Table Morphological characteristics of 13 snow-inhabiting species having elongate or ellipsoidal vegetative cells with a rounded posterior end, in the genus *Chloromonas* sensu Ettl. (DOCX)

S1 Text Taxonomic treatment of the strain UTEX SNO74. (DOCX)

S2 Text Key to vegetative cells of snow-inhabiting species of *Chloromonas* sensu Ettl. (DOCX)
