# Supplementary figures and images for: Taxonomic revision of Chloromonas nivalis (Volvocales, Chlorophyceae) strains, with the new description of two snow-inhabiting Chloromonas species
Source: PLoS One. 2018 Mar 23;13(3):e0193603. doi: 10.1371/journal.pone.0193603 (PMC5865719; doi:10.1371/journal.pone.0193603)

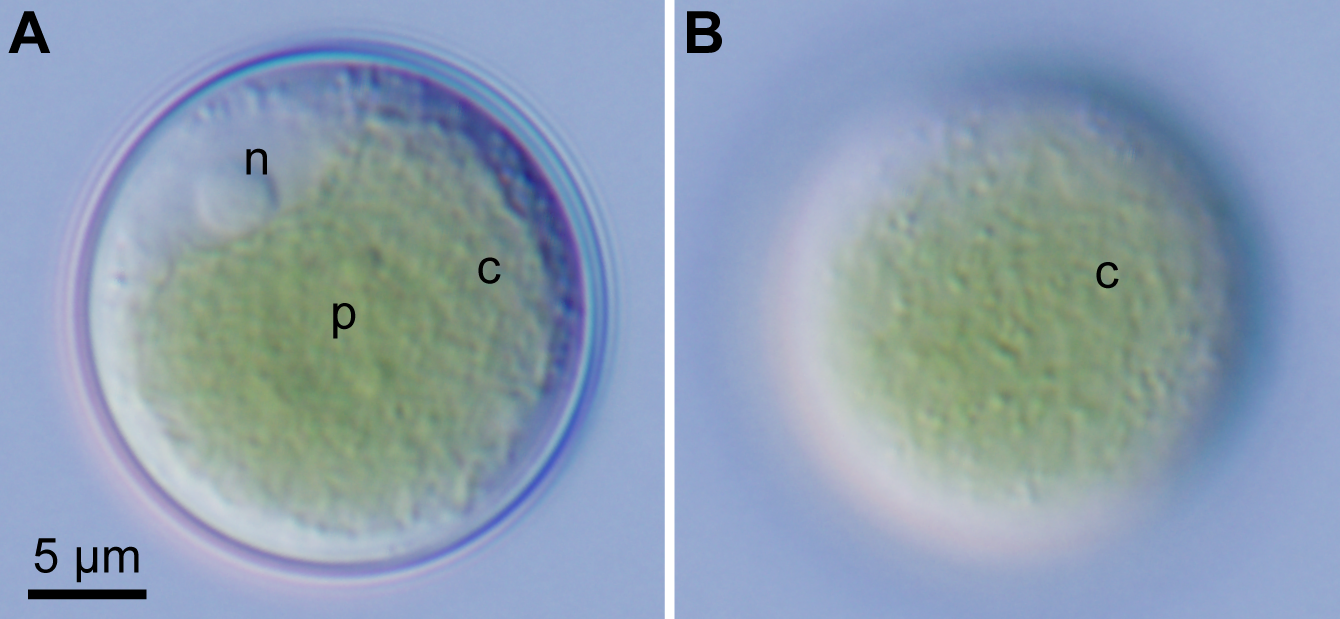

Supplement: S1 Fig — Abbreviations: c, chloroplast; n, nucleus; p, pyrenoid. (A) Optical section focused on a pyrenoid. (B) Surface view. The strain [formerly designated as Chloromonas nivalis (Chodat) Hoham et Mullet] was not used in course of this study since the strain might be replaced with contamination by the species of the genus Trebouxia (see S1 Text; S2 Table). (TIF) [file pone.0193603.s001.tif]

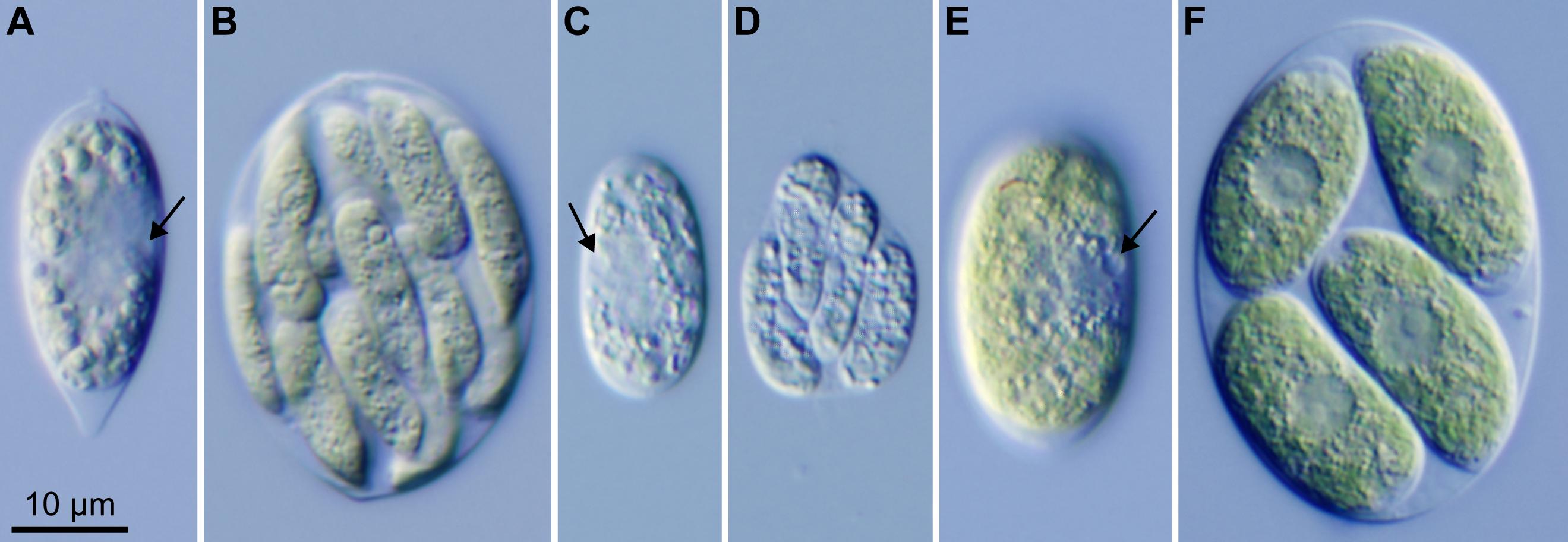

Supplement: S2 Fig — All at identical magnification. Arrows in A, C, E indicate position of each contractile vacuole originating from the parent cell. (A, B) C. nivalis (Chodat) Hoham et Mullet strain UTEX SNO71. (A) Immediately prior to the first transverse division. (B) Sixteen daughter cells within the parental cell wall. Note that only 12 of the 16 cells are recognized. (C, D) C. hoshawii Matsuzaki et al. sp. nov. strain UTEX SNO66. (C) Immediately prior to the first transverse division. (D) Four daughter cells within the parental cell wall. (E, F) C. remiasii Matsuzaki et al. sp. nov. strain CCCryo 005–99. (E) Immediately prior to the first transverse division. (F) Four daughter cells within the parental cell wall. (TIF) [file pone.0193603.s002.tif]

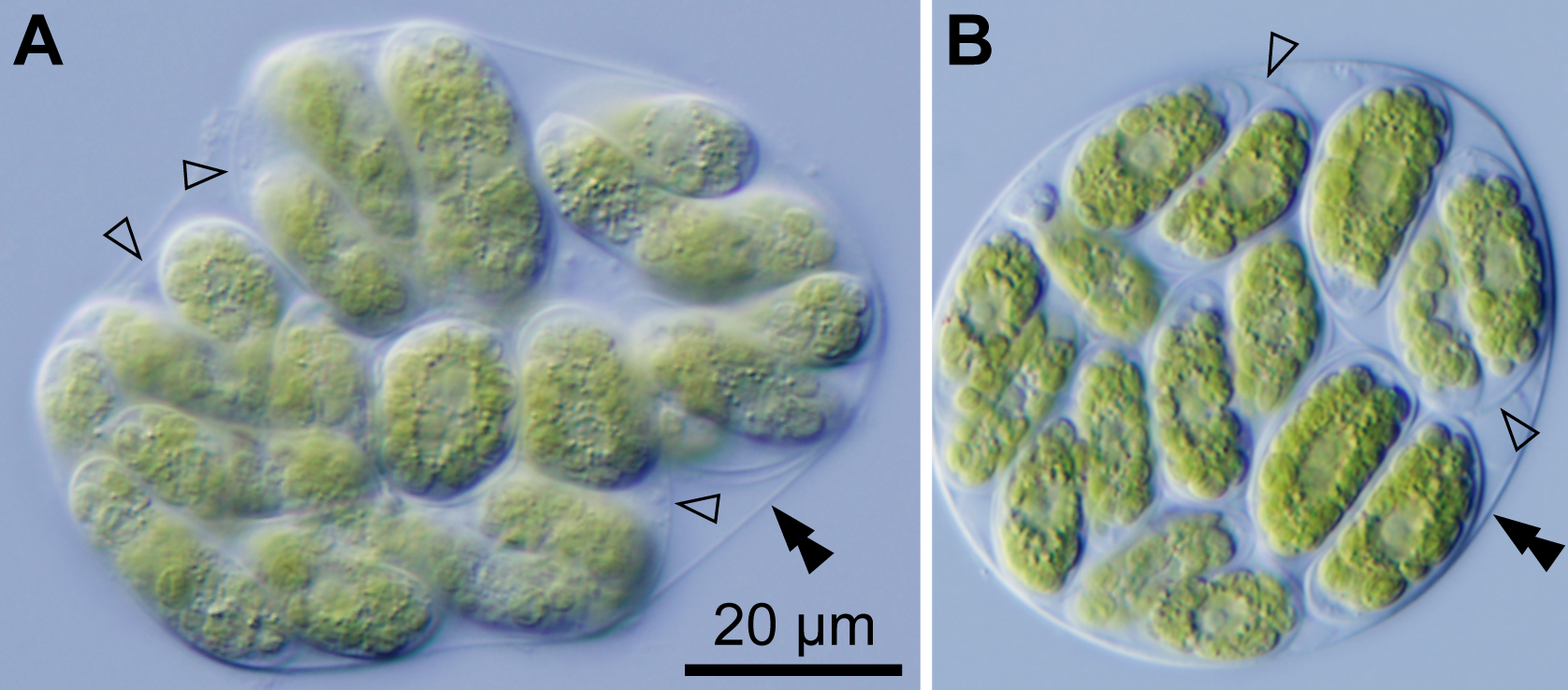

Supplement: S3 Fig — Aggregates result from repeated divisions of daughter cells retained in parental cell walls (double arrowhead). Open arrowhead indicates a daughter cell wall surrounding offspring of a daughter cell. All at the identical magnification. (A) Strain CCCryo 005–99 after 7 days in liquid AF-6 medium. (B) Strain CCCryo 047–99 after 3 months on 1.5% agar slant of AF-6. (TIF) [file pone.0193603.s003.tif]

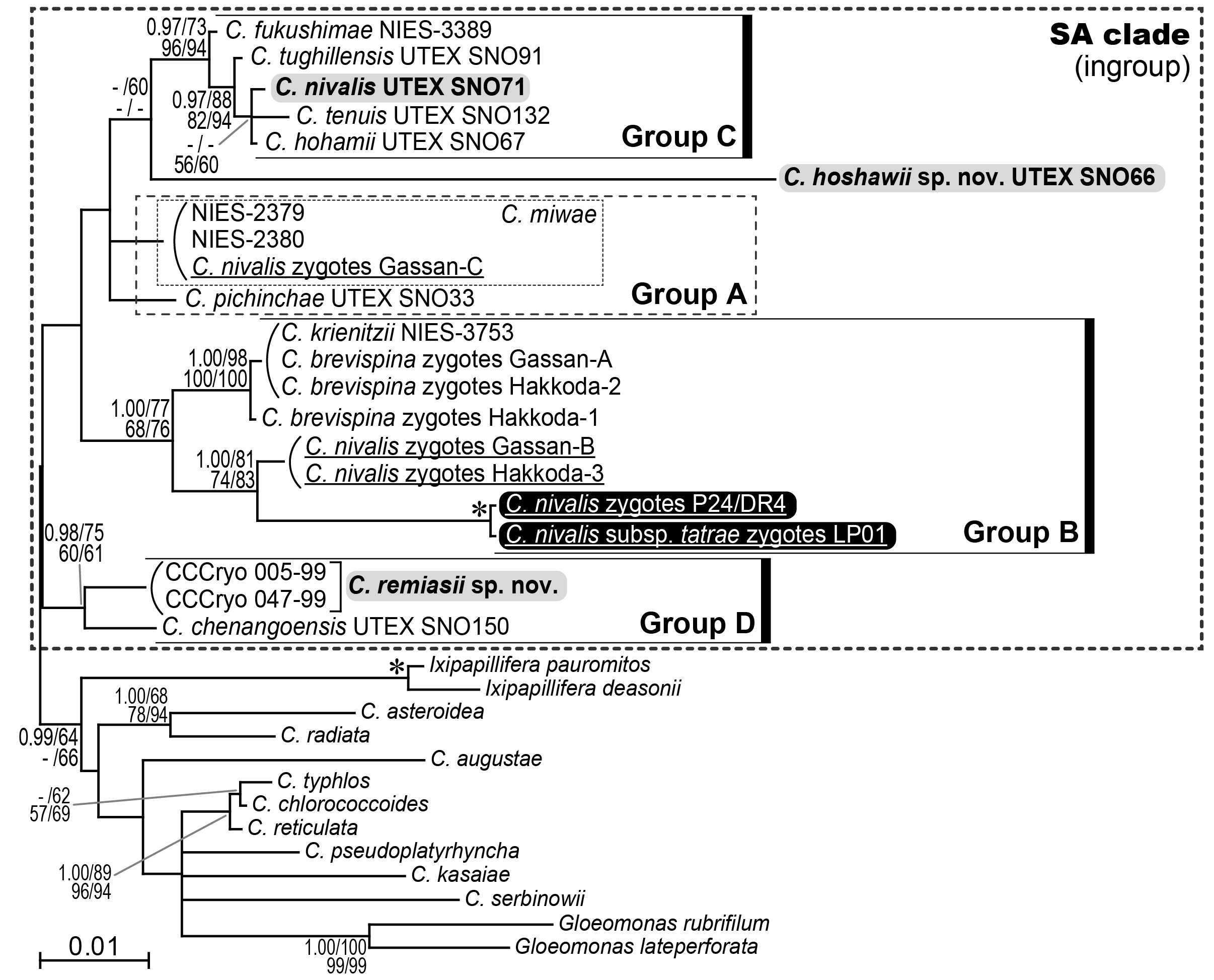

Supplement: S4 Fig — C. nivalis zygote specimens (Field-collected samples) are underlined, and the Austrian C. nivalis zygote specimen (P24/DR4 [19]) and the Slovak C. nivalis subsp. tatrae zygote specimen (LP01 [20]) are shadowed in black. Groups A–D are as indicated in Fig 4. The corresponding posterior probabilities (PP, 0.95 or more) are shown at the top left. Numbers shown in top right, bottom left and bottom right indicate bootstrap values (BV, 50% or more) from maximum likelihood (ML), maximum parsimony (MP) and neighbor-joining (NJ) analyses. Asterisk indicates 1.00 PP in BI and 100% BV in ML, MP, and NJ analyses. (TIF) [file pone.0193603.s004.tif]

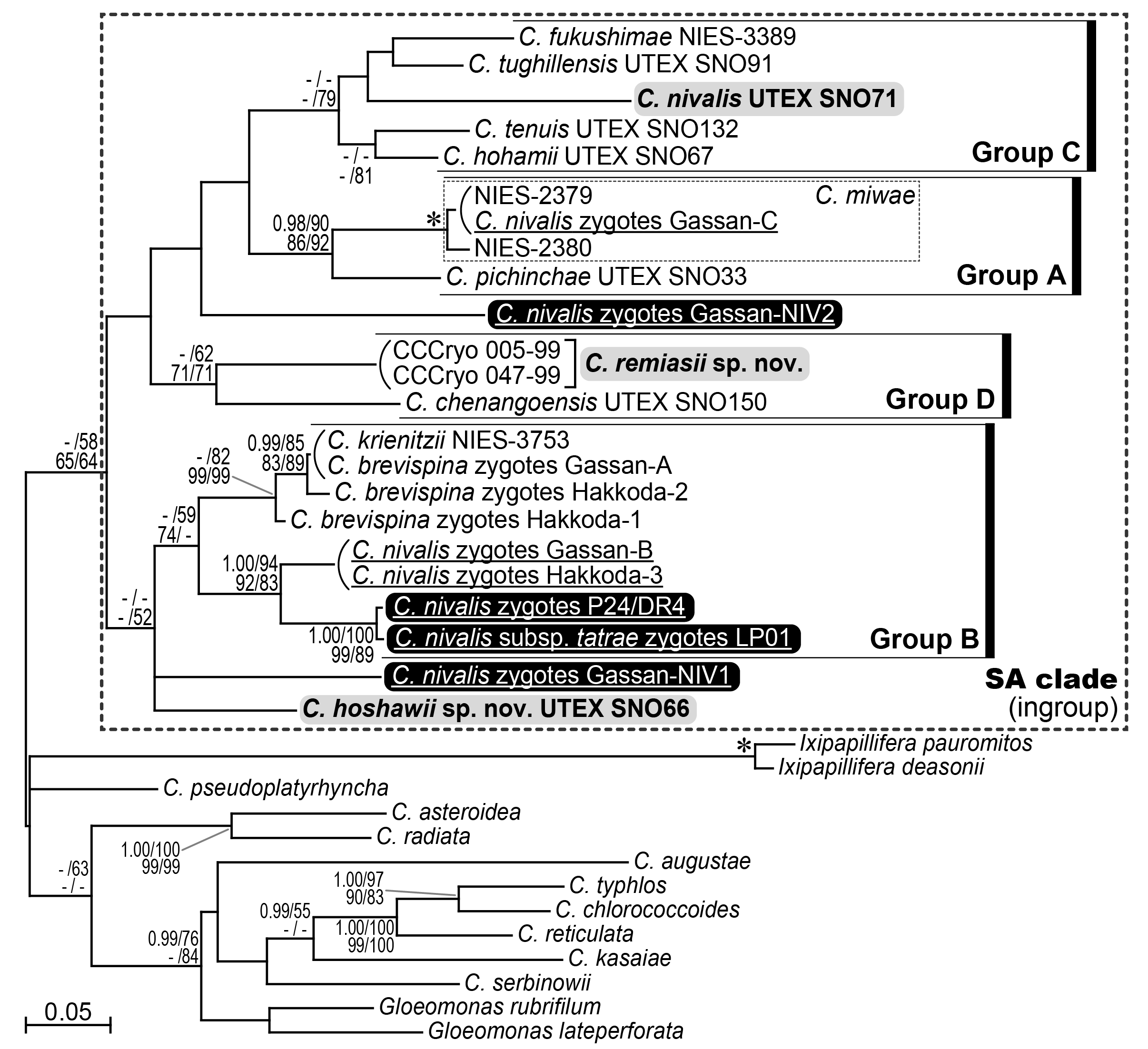

Supplement: S5 Fig — C. nivalis zygote specimens (Field-collected samples) are underlined, and the Austrian and Japanese C. nivalis zygote specimens examined in the previous studies (P24/DR4 [19,20], and Gassan-NIV1 and Gassan-NIV2 [41], respectively) and the Slovak C. nivalis subsp. tatrae zygote specimen (LP01 [20]) are shadowed in black. Groups A–D are as in Fig 4. The corresponding posterior probabilities (PP, 0.95 or more) are shown at the top left. Numbers shown in top right, bottom left and bottom right indicate bootstrap values (BV, 50% or more) from maximum likelihood (ML), maximum parsimony (MP) and neighbor-joining (NJ) analyses. Asterisk indicates 1.00 PP in BI and 100% BV in ML, MP and NJ analyses. (TIF) [file pone.0193603.s005.tif]

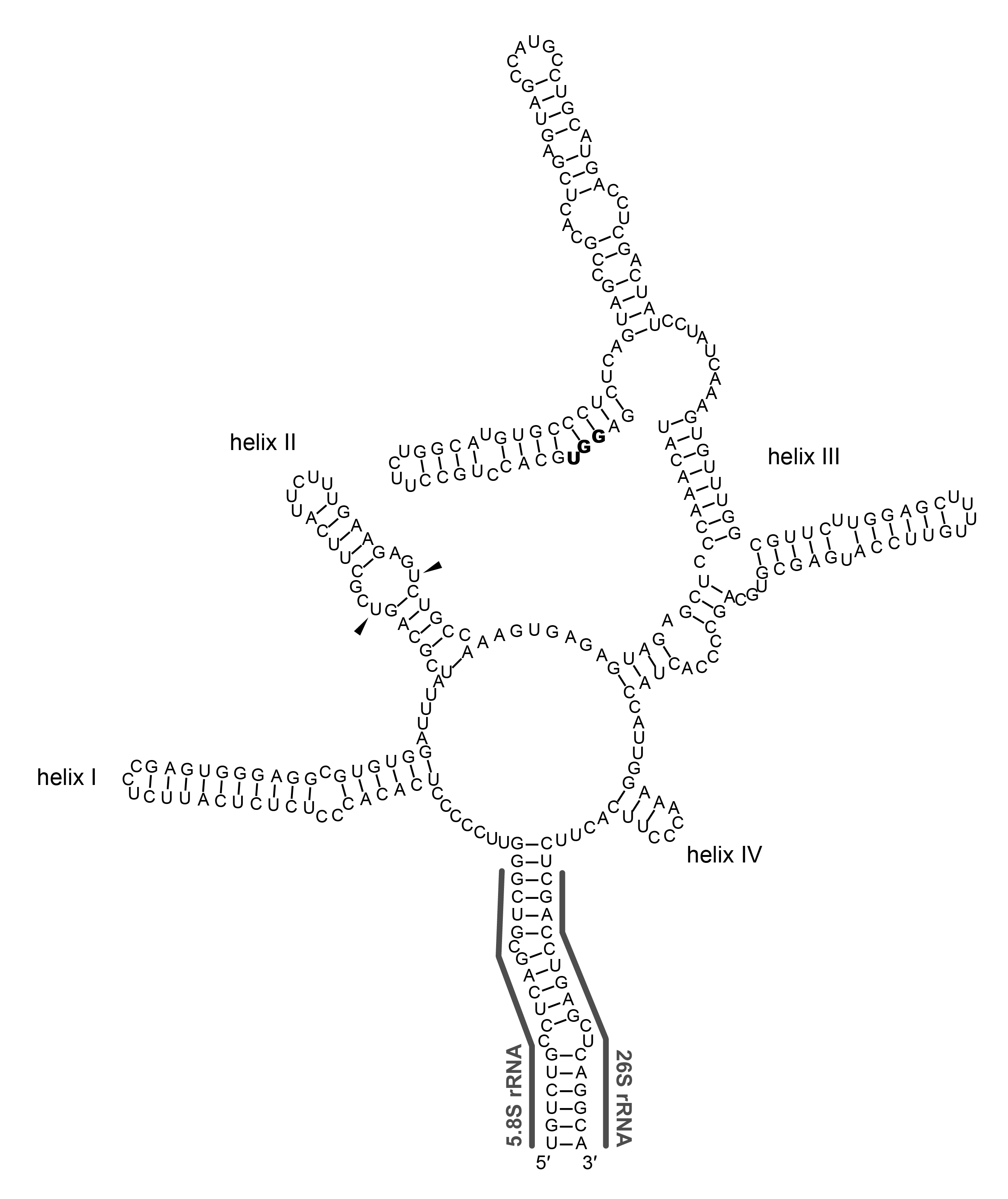

Supplement: S6 Fig — The 3′ end of the 5.8S ribosomal RNA (rRNA) and the 5′ end of the 26S rRNA are shown (DDBJ/ENA/GenBank accession number: HQ404862). The sequence from C. remiasii strains CCCryo 005–99 is identical to that from CCCryo 047–99 (LC360496). Note U-U mismatch in helix II (arrowheads) and the YGGY motif on the 5′ side near the apex of helix III (boldface), common structural hallmarks of eukaryotic nuclear rDNA ITS2 secondary structures [47,50]. (TIF) [file pone.0193603.s006.tif]

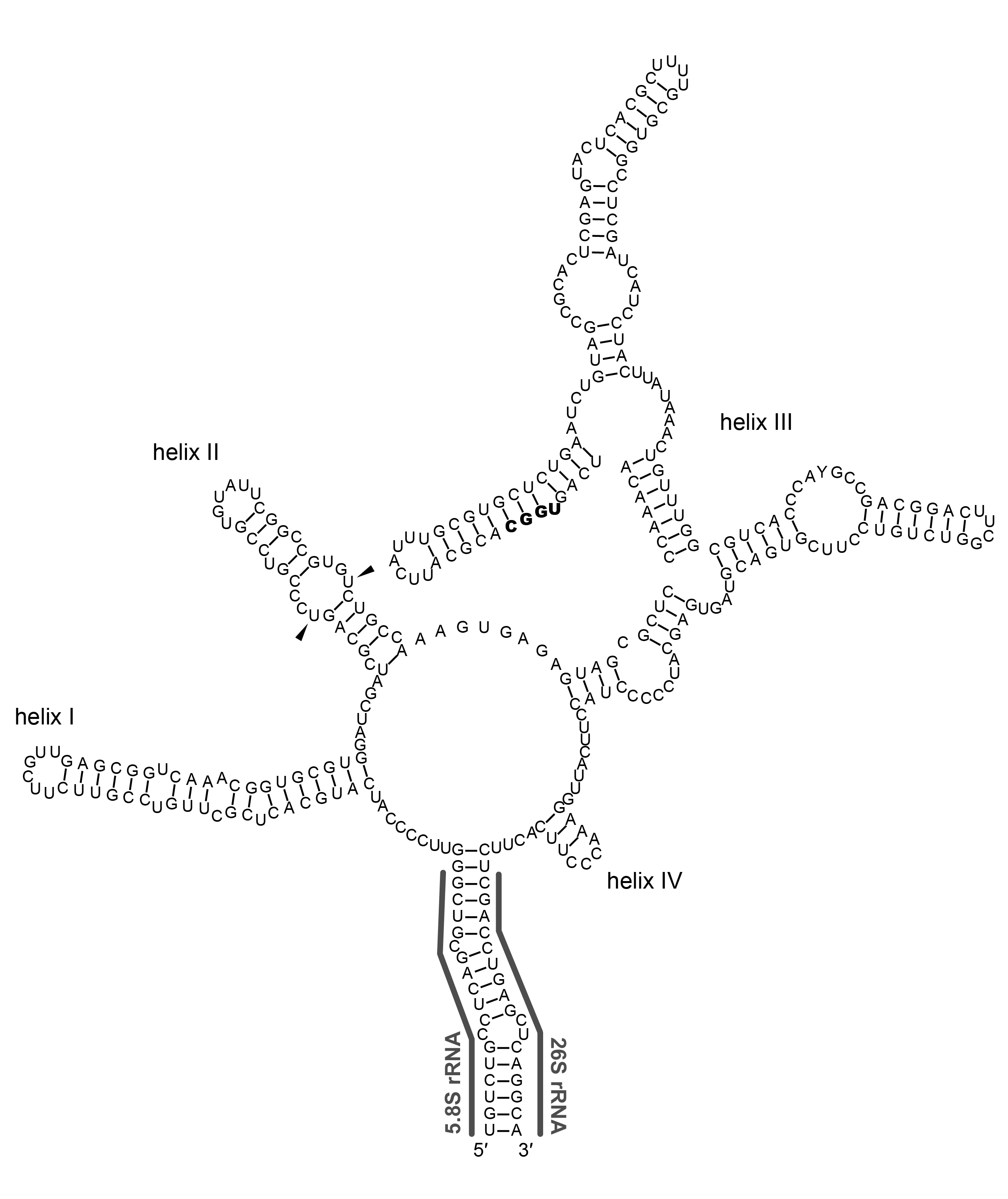

Supplement: S7 Fig — The 3′ end of the 5.8S ribosomal RNA (rRNA) and the 5′ end of the 26S rRNA are shown (DDBJ/ENA/GenBank accession number: LC360497). Note U-U mismatch in helix II (arrowheads) and the YGGY motif on the 5′ side near the apex of helix III (boldface), common structural hallmarks of eukaryotic nuclear rDNA ITS2 secondary structures [47,50]. (TIF) [file pone.0193603.s007.tif]
