# Supplementary material for: Taxonomic revision of Chloromonas nivalis (Volvocales, Chlorophyceae) strains, with the new description of two snow-inhabiting Chloromonas species
Source: PLoS One. 2018 Mar 23;13(3):e0193603. doi: 10.1371/journal.pone.0193603 (PMC5865719; doi:10.1371/journal.pone.0193603)
Supplement: S1 Table — (DOCX) [file pone.0193603.s008.docx]

**S1 Table. Strains examined in this study.**

| Taxon | Designation | Origin | Collection date | Reference |
| --- | --- | --- | --- | --- |
| *Chloromonas hoshawii* sp. nov. | UTEX SNO66 | Mt. Lemmon, Arizona, USA | 1987 Apr 26 | [1,2] |
| *Chloromonas nivalis* | UTEX SNO71 | Mt. Lemmon, Arizona, USA | 1987 Apr 26 | [2] |
| *Chloromonas remiasii* sp. nov. | CCCryo 005-99 | Reuschhalvøya, Spitsbergen, Svalbard, Norway | 1999 Sep 2 | [3–5] |
|  | CCCryo 047-99 | Reuschhalvøya, Spitsbergen, Svalbard, Norway | 1999 Sep 29 | [3–5] |
| *Trebouxia* sp. | UTEX SNO74^1^ | Mt. Lemmon, Arizona, USA | 1987 Apr 26 | [2] |

^1^For species identification of the strain, see S1 Text.

**References**

1. Hoham RW, Bonome TA, Martin CW, Leebens-Mack JH. A combined 18S rDNA and *rbc*L phylogenetic analysis of *Chloromonas* and *Chlamydomonas* (Chlorophyceae, Volvocales) emphasizing snow and other cold-temperature habitats. J Phycol. 2002;38: 1051–1064. doi: 10.1046/j.1529-8817.2002.t01-1-01227.x.

2. UTEX Culture Collection of Algae [Internet]. Texas: The University of Texas at Austin; c2017 [cited 2017 Jan 26]. Available from: <https://utex.org/>.

3. Leya T. Feldstudien und genetische Untersuchungen zur Kryophilie der Schneealgen Nordwestspitzbergens [dissertation]. Berlin: Humboldt-Universität zu Berlin; 2004. German.

4. Remias D, Karsten U, Lütz C, Leya T. Physiological and morphological processes in the alpine snow alga *Chloromonas nivalis* (Chlorophyceae) during cyst formation. Protoplasma. 2010;243: 73–86. doi: 10.1007/s00709-010-0123-y. PubMed PMID: 20229328.

5. Culture Collection of Cryophilic Algae [Internet]. Brandenburg: The Fraunhofer Institute for Cell Therapy and Immunology; c2016 [cited 2017 Jan 26]. Available from: <http://cccryo.fraunhofer.de/web/infos/welcome/>.
