# Supplementary material for: Taxonomic revision of Chloromonas nivalis (Volvocales, Chlorophyceae) strains, with the new description of two snow-inhabiting Chloromonas species
Source: PLoS One. 2018 Mar 23;13(3):e0193603. doi: 10.1371/journal.pone.0193603 (PMC5865719; doi:10.1371/journal.pone.0193603)
Supplement: S2 Table — (DOCX) [file pone.0193603.s009.docx]

**S2 Table. BLASTn results using two gene sequences of the four strains as queries against nucleotide collection.**

| Query (length) | Top hit (accession number) | Total Score | Query coverage | E value | Identity |
| --- | --- | --- | --- | --- | --- |
| UTEX SNO74 |  |  |  |  |  |
| 18S rDNA (1752 bp) | *Trebouxia impressa* (Z21551^1^) | 3208 | 1752/1752 (100%) | 0.0 | 1746/1752 (99%) |
| *rbc*L (1128 bp) | *Trebouxia anticipata* (AF189069^2^) | 1960 | 1121/1128 (99%) | 0.0 | 1101/1121 (98%) |
| CCCryo 005-99^3^ |  |  |  |  |  |
| 18S rDNA (1746 bp) | *Chloromonas chenangoensis* (AB906341^4^) | 3123 | 1746/1746 (100%) | 0.0 | 1728/1747 (99%) |
| *rbc*L (1128 bp) | *Chloromonas tughillensis* (LC012747^5^) | 1652 | 1128/1128 (100%) | 0.0 | 1051/1129 (93%) |
| UTEX SNO66 |  |  |  |  |  |
| 18S rDNA (1748 bp) | *Chloromonas fukushimae* (AB906342^6^) | 2844 | 1746/1746 (100%) | 0.0 | 1683/1752 (96%) |
| *rbc*L (1128 bp) | *Chloromonas pseudoplatyrhyncha* (LC012752) | 1613 | 1128/1128 (100%) | 0.0 | 1043/1128 (92%) |
| UTEX SNO71 |  |  |  |  |  |
| 18S rDNA (1746 bp) | *Chloromonas hohamii* (AB906344) | 3214 | 1746/1746 (100%) | 0.0 | 1744/1746 (99%) |
| *rbc*L (1128 bp) | *Chloromonas tughillensis* (LC012747^5^) | 1696 | 1128/1128 (100%) | 0.0 | 1059/1129 (94%) |

The results were obtained on 2018 Feb 02. Uncultured/environmental sample sequences were excluded from the results. Abbreviations: *rbc*L, RuBisCO large subunit gene; rDNA, ribosomal DNA.

^1^ The nucleotide sequence was obtained from the strain UTEX 892, the authentic strain of *T. impressa* Ahmadjian [1,2].

^2^ The nucleotide sequence was obtained from the strain UTEX 903, the authentic strain of *T. anticipata* Ahmadjian *ex* Archibald [2].

^3^ Sequences of 18S rDNA and *rbc*L from the strains CCCryo 005-99 and CCCryo 047-99 were identical.

^4^ The nucleotide sequence was obtained from the strain UTEX SNO150, the authentic strain of *C. chenangoensis* Hoham et al. [3,4].

^5^ The nucleotide sequence was obtained from the strain UTEX SNO91, the authentic strain of *C. tughillensis* Hoham et al. [3,4].

^6^ The nucleotide sequence was obtained from the strain NIES-3389, the authentic strain of *C. fukushimae* Matsuzaki et Nozaki [4].

**References**

1. Ahmadjian V. Some new and interesting species of *Trebouxia*, a genus of lichenized algae. Am J Bot. 1960;47: 677–683.

2. Archibald PA. *Trebouxia* de Pulmaly (Chlorophyceae, Chlorococcales) and *Pseudotrebouxia* gen. nov. (Chlorophyceae, Chlorosarcinales). Phycologia. 1975;14: 125–137. doi: 10.2216/i0031-8884-14-3-125.1.

3. Hoham RW, Berman JD, Rogers HS, Felio JH, Ryba JB, Miller PR. Two new species of green snow algae from Upstate New York, *Chloromonas chenangoensis* sp. nov. and *Chloromonas tughillensis* sp. nov. (Volvocales, Chlorophyceae) and the effects of light on their life cycle development. Phycologia. 2006;45: 319–330. doi: 10.2216/04-103.1.

4. Matsuzaki R, Hara Y, Nozaki H. A taxonomic study of snow *Chloromonas* species (Volvocales, Chlorophyceae) based on light and electron microscopy and molecular analysis of cultured material. Phycologia. 2014;53: 293–304. doi: 10.2216/14-3.1.
