# Supplementary material for: Taxonomic revision of Chloromonas nivalis (Volvocales, Chlorophyceae) strains, with the new description of two snow-inhabiting Chloromonas species
Source: PLoS One. 2018 Mar 23;13(3):e0193603. doi: 10.1371/journal.pone.0193603 (PMC5865719; doi:10.1371/journal.pone.0193603)
Supplement: S3 Table — (DOCX) [file pone.0193603.s010.docx]

**S3 Table.** **Taxa/specimens/strains in the present molecular analyses (Figs 4 and 5; S4 and S5 Figs) and DDBJ/ENA/GenBank accession numbers of the five genes.**

| Taxon | Specimen/strain  designation | Accession number | | | | |
| --- | --- | --- | --- | --- | --- | --- |
|  |  | 18S rDNA | 26S rDNA | *atp*B | *psa*B | *rbc*L |
| Snow-inhabiting species  (ingroup) | | | | | | |
| *C. brevispina* | Gassan-A^1^ | LC012709 | LC060471 | LC012717 | LC012725 | LC012733 |
|  | Hakkoda-1^2^ | LC012710 | LC060472 | LC012718 | LC012726 | LC012734 |
|  | Hakkoda-2^2^ | LC012711 | LC060473 | LC012719 | LC012727 | LC012735 |
| *C. chenangoensis* | UTEX^3^ SNO150  (authentic) | AB906341 | LC360468^4^ | AB906360 | AB906371 | LC012736 |
| *C. fukushimae* | NIES^5^-3389  (authentic) | AB906342 | AB906352 | AB906361 | AB906372 | LC012738 |
| *C. hohamii* | UTEX SNO67 | AB906344 | AB906354 | AB906363 | AB906374 | AB434265,  LC012742 |
| *C. hoshawii* sp. nov. | UTEX SNO66 | AB906345 | LC360469^4^ | AB906364 | AB906375 | AB434272 |
| *C. krienitzii* | NIES-3753 (authentic) | LC012712 | LC060474 | LC012720 | LC012728 | LC012740 |
| *C. miwae* | NIES-2379 | AB906350 | LC060476 | AB906369 | AB906380 | AB434271 |
|  | NIES-2380 | AB906351 | LC060477 | AB906370 | AB906381 | AB434270 |
| *C. nivalis* | Gassan-B^1^ | LC012714 | LC060478 | LC012722 | LC012730 | LC012743 |
|  | Gassan-C^1^ | LC012715 | LC060479 | LC012723 | LC012731 | LC012744 |
|  | Gassan-NIV1^6^ |  |  |  |  | AB434274 |
|  | Gassan-NIV2^6^ |  |  |  |  | AB434275 |
|  | Hakkoda-3^2^ | LC012716 | LC060480 | LC012724 | LC012732 | LC012745 |
|  | P24/DR4^7^ | GU117576 |  |  |  | KY499616 |
|  | UTEX SNO71 | LC360465^4^ | LC360470^4^ | LC360484^4^ | LC360488^4^ | LC360492^4^ |
| *C. nivalis* subsp. *tatrae* | LP01^8^ | KY499614 |  |  |  | KY499615 |
| *C. pichinchae* | UTEX SNO33 | AB906346 | LC060481 | AB906365 | AB906376 | AB434266,  LC012746 |
| *C. remiasii* sp. nov. | CCCryo^9^ 005-99 | LC360466^4^ | LC360471^4^ | LC360485^4^ | LC360489^4^ | LC360493^4^ |
|  | CCCryo 047-99 | LC360467^4^ | LC360472^4^ | LC360486^4^ | LC360490^4^ | LC360494^4^ |
| *C. tenuis* | UTEX SNO132  (authentic) | AB906347 | AB906355 | AB906366 | AB906377 | AB434263 |
| *C. tughillensis* | UTEX SNO91  (authentic) | AB906348 | AB906356 | AB906367 | AB906378 | LC012747 |
| Mesophilic species  (outgroup) | | | | | | |
| *C. asteroidea* | SAG^10^ 11-47^11^ | U70783 | LC360473^4^ | AB084808 | AB084342 | AB022225 |
| *C. augustae* | SAG 5.73^11^ | AJ410452 | LC360474^4^ | AB504757 | AB504769 | AB504764 |
| *C. chlorococcoides* | SAG 15.82 (authentic) | AJ410449, AB624555 | AB906359 | AB624580 | AB624595 | LC361432^4^ |
| *C. kasaiae* | NIES-2862 (authentic) | AB734109 | LC360475^4^ | AB734110 | AB734111 | LC012751 |
| *C. pseudoplatyrhyncha* | NIES-2563 | AB548689 | LC360476^4^ | AB548690 | AB548691 | LC012752 |
| *C. radiata* | UTEX 966^11^ | U57697 | LC360477^4^ | AB084311 | AB084345 | AJ001878 |
| *C. reticulata* | SAG 29.83  (= UTEX 1970^11^) | U70791, AB624560 | AF395508 | AB084312 | AB084346, AB084347 | AB022534 |
| *C. serbinowii* | UTEX 492^11^  (= SAG 11.84) | U70795, AB624568, AB624569 | LC360478^4^ | AB084317 | AB084354 | AJ001879 |
| *C. typhlos* | SAG 26.86  (= UTEX 1969) | AB624566 | LC360479^4^ | AB084307 | AB084341 | AB022228 |
| *G. lateperforata* | NIES-464 | AB504779 | LC360480^4^ | AB504761 | AB504773 | AB504768 |
| *G. rubrifilum* | SAG 3.85^12^ | AJ410455 | LC360481^4^ | AB504758 | AB504770 | AB504765 |
| *I. deasonii* | SAG 46.72 (authentic) | AJ410446 | LC360482^4^ | AB101503 | AB101514 | AB101508 |
| *I. pauromitos* | NIES-3707 (authentic) | LC057290 | LC360483^4^ | LC360487^4^ | LC360491^4^ | LC360495^4^ |

Abbreviations: *atp*B, ATP synthase beta subunit gene; *C.*, *Chloromonas*; *G.*, *Gloeomonas*; *I.*, *Ixipapillifera*; *psa*B, P700 chlorophyll *a* apoprotein A2 gene; *rbc*L, RuBisCO large subunit gene; rDNA, ribosomal DNA.

^1^Specimen of zygotes collected from Mt. Gassan, Japan [1].

^2^Specimen of zygotes collected from Mt. Hakkoda, Japan [1].

^3^Culture Collection of Algae at the University of Texas at Austin [2,3].

^4^Sequenced in the present study.

^5^Microbial Culture Collection at the National Institute for Environmental Studies [4,5].

^6^Specimen of zygotes collected from Mt. Gassan, Japan [6].

^7^Specimen of zygotes collected from Austrian Alps [7].

^8^Specimen of zygotes collected from the High Tatra Mountains, Slovakia [8]

^9^Culture Collection of Cryophilic Algae at the Fraunhofer Institute for Cell Therapy and Immunology [9].

^10^Sammlung von Algenkulturen at the University of Göttingen [10,11].

^11^Epitype proposed by Pröschold et al. [12].

^12^Epitype of *Chloromonas rubrifilum* proposed by Pröschold et al. [12].

**References**

1. Matsuzaki R, Kawai-Toyooka H, Hara Y, Nozaki H. Revisiting the taxonomic significance of aplanozygote morphologies of two cosmopolitan snow species of the genus *Chloromonas* (Volvocales, Chlorophyceae). Phycologia. 2016;54: 491–502. doi: 10.2216/15-33.1.

2. Starr RC, Zeikus JA. UTEX – the culture collection of algae at the University of Texas at Austin. J Phycol. 1993;29 Suppl 2: 1–106. doi: 10.1111/j.0022-3646.1993.00001.x.

3. UTEX Culture Collection of Algae [Internet]. Texas: The University of Texas at Austin; c2017 [cited 2017 Jan 26]. Available from: <https://utex.org/>.

4. Kawachi M, Ishimoto M, Mori F, Yumoto K, Sato M, Noël M-H. MCC-NIES. List of Strains, 9th Edition [DVD]. Tsukuba: National Institute for Environmental Studies; 2013.

5. Microbial Culture Collection at National Institute for Environmental Studies [Internet]. Ibaraki: The National Institute for Environmental Studies; c2001 [cited 2017 Oct 25]. Available from: <http://mcc.nies.go.jp/index_en.html>.

6. Muramoto K, Kato S, Shitara T, Hara Y, Nozaki H. Morphological and genetic variation in the cosmopolitan snow alga *Chloromonas nivalis* (Volvocales, Chlorophyceae) from Japanese mountainous area. Cytologia. 2008;73: 91–96. doi: 10.1508/cytologia.73.91.

7. Remias D, Karsten U, Lütz C, Leya T. Physiological and morphological processes in the alpine snow alga *Chloromonas nivalis* (Chlorophyceae) during cyst formation. Protoplasma. 2010;243: 73–86. doi: 10.1007/s00709-010-0123-y. PubMed PMID: 20229328.

8. Procházková L, Remias D, Řezanka T, Nedbalová L. *Chloromonas nivalis* subsp. *tatrae*, subsp. nov. (Chlamydomonadales, Chlorophyta): re-examination of a snow alga from the High Tatra Mountains (Slovakia). Fottea. 2018;18: 1–18. doi: 10.5507/fot.2017.010.

9. Culture Collection of Cryophilic Algae [Internet]. Brandenburg: The Fraunhofer Institute for Cell Therapy and Immunology; c2016 [cited 2017 Jan 26]. Available from: <http://cccryo.fraunhofer.de/web/infos/welcome/>.

10. Schlösser UG. SAG – Sammlung von Algenkulturen at the University of Göttingen. Catalogue of strains 1994. Bot Acta. 1994;107: 113–186. doi: 10.1111/j.1438-8677.1994.tb00784.x.

11. The Culture Collection of Algae at the University of Göttingen [Internet]. Göttingen: The University of Göttingen; c2017 [cited 2017 Oct 25]. Available from: <http://sagdb.uni-goettingen.de/index.php>.

12. Pröschold T, Marin B, Schlösser UG, Melkonian M. Molecular phylogeny and taxonomic revision of *Chlamydomonas* (Chlorophyta). I. Emendation of *Chlamydomonas* Ehrenberg and *Chloromonas* Gobi, and description of *Oogamochlamys* gen. nov. and *Lobochlamys* gen. nov. Protist. 2001;152: 265–300. doi: 10.1078/1434-4610-00068. PubMed PMID: 11822658.
