# Supplementary material for: Taxonomic revision of Chloromonas nivalis (Volvocales, Chlorophyceae) strains, with the new description of two snow-inhabiting Chloromonas species
Source: PLoS One. 2018 Mar 23;13(3):e0193603. doi: 10.1371/journal.pone.0193603 (PMC5865719; doi:10.1371/journal.pone.0193603)
Supplement: S4 Table — (DOCX) [file pone.0193603.s011.docx]

**S4 Table. Primers for amplification and sequencing of P700 chlorophyll *a* apoprotein A2 gene from *Chloromonas remiasii* strains.**

| Designation | Position^1^ | Sequence (5′–3′) |
| --- | --- | --- |
| CCCryo_F | 229–251 | TGGGTGACTGATCCAATTCATGT |
| CCCryo_R | 1205–1186^2^ | AGTTCCTGGGTCATAATCTCG |

^1^Coordinate numbers from the gene of *Chlorella vulgaris* [1].

^2^Reverse primer.

**Reference**

1. Wakasugi T, Nagai T, Kapoor M, Sugita M, Ito M, Ito S, et al. Complete nucleotide sequence of the chloroplast genome from the green alga *Chlorella vulgaris*: The existence of genes possibly involved in chloroplast division. Proc Natl Acad Sci U S A. 1997;94: 5967–5972. PubMed PMID: 9159184; PubMed Central PMCID: PMC20890.
