# Supplementary material for: Taxonomic revision of Chloromonas nivalis (Volvocales, Chlorophyceae) strains, with the new description of two snow-inhabiting Chloromonas species
Source: PLoS One. 2018 Mar 23;13(3):e0193603. doi: 10.1371/journal.pone.0193603 (PMC5865719; doi:10.1371/journal.pone.0193603)
Supplement: S5 Table — (DOCX) [file pone.0193603.s012.docx]

**S5 Table.** **Substitution models applied to respective data matrices of the present phylogenetic analyses (Fig 4; S4 and S5 Figs).**

|  | Substitution model(s) | | |
| --- | --- | --- | --- |
| Data matrix/Figure No. | BI | ML | NJ |
| concatenated 18S and 26S rDNA, and *atp*B and *psa*B (first, second codons only) from 27 OTUs/Fig 4 | partitioned into  18S rDNA (GTR+I+G),  26S rDNA (GTR+I+G),  *atp*B (SYM+I+G), and  *psa*B (GTR+I+G) | partitioned into  18S rDNA (K2P+I),  26S rDNA (GTR+G4),  *atp*B (TIM2e+I), and  *psa*B (TNe+I) | TrN+I+G |
| 18S rDNA from 28 OTUs/S4 Fig | GTR+I+G | TN+I | TrN+I+G |
| *rbc*L from 32 OTUs/S5 Fig | partitioned into  first codons (GTR+I+G),  second codons (JC+G), and third codons (GTR+I+G) | partitioned into  first codons (TIM+I),  second codons (JC+I), and third codons (TPM3+G4) | GTR+I+G |

Each substitution model was selected by hierarchical likelihood ratio test using MrModeltest 2.3 [1] for Bayesian inference (BI) or Modeltest 3.7 [2] for neighbor-joining (NJ) analysis, or selected by the Bayesian information criterion using IQ-TREE v. 1.4.3 [3] for maximum likelihood (ML) analysis.

Abbreviations: *atp*B, ATP synthase beta subunit gene; OTUs, operational taxonomic units; *psa*B, P700 chlorophyll *a* apoprotein A2 gene; *rbc*L, RuBisCO large subunit gene; rDNA, ribosomal DNA.

**References**

1. Nylander JAA. MrModeltest 2.3 [software]. 2008 May 22 [cited 2017 Jan 26]. Available from: <https://github.com/nylander/MrModeltest2>.

2. Posada D, Crandall KA. Modeltest: testing the model DNA substitution. Bioinformatics. 1998;14: 817–818. doi: 10.1093/bioinformatics/14.9.817. PubMed PMID: 9918953.

3. Nguyen L-T, Schmidt HA, von Haeseler A, Minh BQ. IQ-TREE: A fast and effective stochastic algorithm for estimating maximum likelihood phylogenies. Mol Biol Evol. 2015;32: 268–274. doi: 10.1093/molbev/msu300. PubMed PMID: 25371430; PubMed Central PMCID: PMC4271533.
