# Supplementary material for: Taxonomic revision of Chloromonas nivalis (Volvocales, Chlorophyceae) strains, with the new description of two snow-inhabiting Chloromonas species
Source: PLoS One. 2018 Mar 23;13(3):e0193603. doi: 10.1371/journal.pone.0193603 (PMC5865719; doi:10.1371/journal.pone.0193603)
Supplement: S6 Table — (DOCX) [file pone.0193603.s013.docx]

**S6 Table. Morphological characteristics of 13 snow-inhabiting species having elongate or ellipsoidal vegetative cells with a rounded posterior end, in the genus *Chloromonas* sensu Ettl [1,2].**

|  | *C. hoshawii* sp. nov. | *C. remiasii* sp. nov. | *C. alpina* | *C. bolyaiana* |
| --- | --- | --- | --- | --- |
| Strain(s) examined | UTEX SNO66 | CCCryo 005-99, CCCryo 047-99 | − | − |
| Cell shape | ellipsoidal to elongate-ovoid | ellipsoidal to spindle-shaped | ellipsoidal to ovoid | ellipsoidal |
| Cell width × cell length (μm) | 4.9–9.3 × 13.8–18.6 | 10.2–15.6× 18.2–30.8 | 4–7 × 9–12 | 46–52 × 52–72 |
| Eyespot | absent | present | present | absent |
| Chloroplast shape | cup-shaped, seemingly composed of angular discs | cup-shaped, seemingly composed of angular discs | parietal, composed of numerous discoid-lobes | asteroid-shaped |
| Longitudinal slit of the chloroplast in the anterior half of the cell | not observed | not observed | not observed | not observed |
| Cell aggregates in old cultures | not observed | observed | − | − |
| Number of zoospores formed within the parental cell | 2 or 4 (rarely 8) | 2 or 4 (rarely 8) | 2 or 4 | 2, 4 or 8 |
| Cyst shape | − | −^1^ | elongate-ellipsoidal with many thick spines on the surface of the cell wall | spherical, having three layered membrane of which the middle layer is ornamented as star-shaped |
| Reference | present study | present study, [3] | [1,2,4] | [1,2,5] |

This table is mainly based on the previous table [9] (see also S2 Text).

^1^A previous study [3] suggested relationship between the strain CCCryo 005-99 and field-collected cysts or zygotes, both of which were collected at the same location in Svalbard. The cysts are spindle-shaped and have several longitudinal, slightly helical ridges on the cell wall extended partially to the poles. Since molecular data of the cysts are not available and sexual reproduction of *C. remiasii* has not been observed, we could not confirm this possible relationship.

**S6 Table.** Extended

| *C. chenangoensis* | *C. fukushimae* | *C. hohamii* | *C. krienitzii* | *C. pichinchae* |
| --- | --- | --- | --- | --- |
| UTEX SNO150,  UTEX SNO143 | NIES-3389, NIES-3390 | UTEX SNO67 | NIES-3753, NIES-3752 | UTEX SNO33 |
| ellipsoidal | elongate-bean- to elongate-kidney-shaped | elongate-ellipsoidal | elongate-bean- to elongate-kidney-shaped | elongate-ovoid to ellipsoidal |
| 7.5–17.5 × 14–25 | 4–9 × 15–23 | 5–10 × 12–25 | 7–11 × 17–24 | 8–15 × 18–26 |
| absent | absent | absent or generally present | absent | absent |
| cup-shaped, seemingly composed of angular discs | laminate parietal with irregular incisions in the dorsal side of the cell, not filling in the posterior end of the protoplast | cup-shaped, apparently composed of elongate- ovoid or elongate- cylindrical platelets | parietal with irregular incisions in the dorsal side of the cell, filling in the posterior end of the protoplast | cup-shaped, seemingly composed of angular discs |
| not observed | not observed | not observed | not observed | not observed |
| not observed | not observed | not observed | not observed | observed |
| generally 2 or 4 | 2, 4 or 8 | 2, 4 or 8 | 2 or 4 | generally 2 or 4 |
| − | − | ellipsoidal, elongate or somewhat fusiform, with longitudinal ridges not spiraled | almost spherical with numerous short spines on the surface of the cell wall, or elongate-ellipsoidal with many thick spines on the surface of the cell wall | ellipsoidal with usually nine to 11 irregular ridges extending over the wall from one cell pole to the other |
| [6,7] | [7] | [7,8] | [9] | [4,7,10] |

**S6 Table.** Extended

| *C. polyptera* | *C. rostafinskii* | *C. tenuis* | *C. tughillensis* |
| --- | --- | --- | --- |
| − | − | UTEX SNO132 | UTEX SNO91, UTEX SNO88, UTEX SNO92 |
| elongate-bean- to elongate-kidney-shaped | elongate-bean- to elongate-kidney-shaped or ellipsoidal | elongate-cylindrical | elongate-ellipsoidal |
| 8–14 × 20–27 | 6.6–13.2 × 11–22.5 | 5–9 × 15–23 | 6–12 × 16–23 |
| generally absent | absent | absent | absent |
| parietal in the dorsal side of the cell | cup-shaped | cup-shaped, seemingly composed of elongate- ovoid or elongate- cylindrical platelets | cup-shaped, seemingly composed of elongate- ovoid or elongate- cylindrical platelets |
| observed | not observed | not observed | not observed |
| observed | − | not observed | observed |
| 2 or 4 | 2 or 4 | generally 2 or 4 | 2, 4, 8 or 16 |
| broadly ellipsoidal with 18–23 slightly spiraled longitudinal ridges | ellipsoidal with thick, warty cell wall | − | spherical |
| [11,12] | [1,2,13,14] | [7] | [6,7] |

**References**

1. Ettl H. Die gattung *Chloromonas* Gobi emend. Wille (*Chlamydomonas* und die nächstverwandten gattungen I). Nova Hedwig Beih. 1970;34: 1–283. German.

2. Ettl H. Chlorophyta I. Phytomonadina*.* In: Ettl H, Gerloff J, Heynig H, Mollenhauer D, editors. Süßwasserflora von Mitteleuropa 9. Stuttgart: G. Fischer Verlag; 1983. p. 1–807. German.

3. Leya T. Feldstudien und genetische Untersuchungen zur Kryophilie der Schneealgen Nordwestspitzbergens [dissertation]. Berlin: Humboldt-Universität zu Berlin; 2004. German.

4. Wille N. Algologische notizen IX–XIV. Nyt Magazin for Naturvidenskaberne. 1903;41: 89–185, with 3 pls. German.

5. Kol E. A new cryobiont of the red snow from Transylvania: *Chlamydomonas bolyaiana* n. sp. Acta Bolyaiana. 1947;1: 132–137.

6. Hoham RW, Berman JD, Rogers HS, Felio JH, Ryba JB, Miller PR. Two new species of green snow algae from Upstate New York, *Chloromonas chenangoensis* sp. nov. and *Chloromonas tughillensis* sp. nov. (Volvocales, Chlorophyceae) and the effects of light on their life cycle development. Phycologia. 2006;45: 319–330. doi: 10.2216/04-103.1.

7. Matsuzaki R, Hara Y, Nozaki H. A taxonomic study of snow *Chloromonas* species (Volvocales, Chlorophyceae) based on light and electron microscopy and molecular analysis of cultured material. Phycologia. 2014;53: 293–304. doi: 10.2216/14-3.1.

8. Hoham RW, Mullet JE, Roemer SC. The life history and ecology of the snow alga *Chloromonas polyptera* comb. nov. (Chlorophyta, Volvocales). Can J Bot. 1983;61: 2416–2429. doi: 10.1139/b83-266.

9. Matsuzaki R, Kawai-Toyooka H, Hara Y, Nozaki H. Revisiting the taxonomic significance of aplanozygote morphologies of two cosmopolitan snow species of the genus *Chloromonas* (Volvocales, Chlorophyceae). Phycologia. 2015;54: 491–502. doi: 10.2216/15-33.1.

10. Hoham RW. The life history and ecology of the snow alga *Chloromonas pichinchae* (Chlorophyta, Volvocales). Phycologia. 1975;14: 213–226. doi: 10.2216/i0031-8884-14-4-213.1.

11. Ling HU, Seppelt RD. Snow algae of the Windmill Islands, continental Antarctica. 3. *Chloromonas polyptera* (Volvocales, Chlorophyta). Polar Biol. 1998;20: 320–324. doi: 10.1007/s003000050309.

12. Remias D, Wastian H, Lütz C, Leya T. Insights into the biology and phylogeny of *Chloromonas polyptera* (Chlorophyta), an alga causing orange snow in maritime Antarctica. Antarct Sci. 2013;25: 648–656. doi: 10.1017/S0954102013000060.

13. Starmach K, Kawecka B. The yellowish-green snow in the Valley Za Mnichem in the Tatra Mountains. Limnological investigation in the Tatra Mountains and Dunajec River Basin. Komitetu Zagospodarowania Ziem Górkich PAN. 1965;11: 75–80.

14. Kawecka B. Biology and ecology of snow algae. 3. Sexual reproduction in *Chloromonas rostafiński* (Starmach et Kawecka) Gerloff et Ettl (Chlorophyta, Volvocales). Acta Hydrobiologica. 1983/1984;25/26: 281–285.
