# Supplementary material for: Taxonomic revision of Chloromonas nivalis (Volvocales, Chlorophyceae) strains, with the new description of two snow-inhabiting Chloromonas species
Source: PLoS One. 2018 Mar 23;13(3):e0193603. doi: 10.1371/journal.pone.0193603 (PMC5865719; doi:10.1371/journal.pone.0193603)
Supplement: S1 Text — (DOCX) [file pone.0193603.s014.docx]

**S1 Text. Taxonomic treatment of the strain UTEX SNO74.**

The strain UTEX SNO74 (= CU565J in [1]) designated as *Chloromonas nivalis* (Chodat) Hoham et Mullet (Volvocales, Chlorophyceae) was used for light microscopic observation of asexual reproductive cell morphology [1]. The present light microscopy (S1 Fig) demonstrated that the vegetative cells of the strain were nonmotile and coccoid in shape, having a massive chloroplast. The chloroplast possessed an indistinct pyrenoid (S1A Fig) and lacked any incisions on the surface (S1B Fig). These morphological characteristics are not consistent with both traditional [2,3] and recently revised [4] concepts of the genus *Chloromonas* Gobi. Besides, Blastn results using the sequences of nuclear-encoded 18S ribosomal DNA and the chloroplast-encoded RuBisCO large subunit gene from the strain UTEX SNO74 (DDBJ/ENA/GenBank accession number, LC360463 and LC360464, respectively) indicated that the strain is phylogenetically close to the species of the genus *Trebouxia* Puymaly (Trebouxiales, Trebouxiophyceae) (S2 Table). Thus, we concluded that the strain UTEX SNO74 has been replaced with *Trebouxia* sp.

**References**

1. Hoham RW, Berman JD, Rogers HS, Felio JH, Ryba JB, Miller PR. Two new species of green snow algae from Upstate New York, *Chloromonas chenangoensis* sp. nov. and *Chloromonas tughillensis* sp. nov. (Volvocales, Chlorophyceae) and the effects of light on their life cycle development. Phycologia. 2006;45: 319–330. doi: 10.2216/04-103.1.

2. Ettl H. Die gattung *Chloromonas* Gobi emend. Wille (*Chlamydomonas* und die nächstverwandten gattungen I). Nova Hedwigia Beih. 1970;34: 1–283. German.

3. Ettl H. Chlorophyta I. Phytomonadina. In: Ettl H, Gerloff J, Heynig H, Molenhauer D, editors. Süßwasserflora von Mitteleuropa 9. Stuttgart: G. Fischer Verlag; 1983. p. 1–807. German.

4. Pröschold T, Marin B, Schlösser UG, Melkonian M. Molecular phylogeny and taxonomic revision of *Chlamydomonas* (Chlorophyta). I. Emendation of *Chlamydomonas* Ehrenberg and *Chloromonas* Gobi, and description of *Oogamochlamys* gen. nov. and *Lobochlamys* gen. nov. Protist. 2001;152: 265–300. doi: 10.1078/1434-4610-00068. PubMed PMID: 11822658.
