# Supplementary material for: Taxonomic revision of Chloromonas nivalis (Volvocales, Chlorophyceae) strains, with the new description of two snow-inhabiting Chloromonas species
Source: PLoS One. 2018 Mar 23;13(3):e0193603. doi: 10.1371/journal.pone.0193603 (PMC5865719; doi:10.1371/journal.pone.0193603)
Supplement: S2 Text — (DOCX) [file pone.0193603.s015.docx]

**S2 Text. Key to vegetative cells of snow-inhabiting species of *Chloromonas* sensu Ettl [1,2].**

The key is mainly based on the previous key [3]. Species examined using cultured material are marked with asterisks (on the basis of [2–9] and the present study).

1. Cell inverted teardrop-shaped with a prominent posterior end ……… *C. nivalis**

1. Cell not inverted teardrop-shaped with a prominent posterior end ……………………… 2

2. Cell not elongate or ellipsoidal ………………………………………… 3

2. Cell elongate or ellipsoidal …………………………………………………………… 6

3. Cell with prominent anterior papilla ………………………………………… 4

3. Cell without prominent anterior papilla ………………………………………… 5

4. Two contractile vacuoles positioned near the base of flagella; anterior papilla without concave top face; maximum cell length less than 15 μm ………………………… *C. andreii*

4. Four contractile vacuoles positioned near the base of flagella; anterior papilla with concave top face; maximum cell length more than 15 μm ………………………… *C. rubroleosa*

5. Cell ovoid or pyriform …………………………………………… *C. brevispina*

5. Cell almost spherical …………………………………………… *C. miwae**

6. Chloroplast asteroid-shaped; maximum cell length more than 40 μm

……………………………………………………………… *C. bolyaiana*

6. Chloroplast not asteroid-shaped; maximum cell length less than 40 μm

……………………………………………………………………………… 7

7. Cell elongate kidney to elongate bean shaped ………………………………………… 8

7. Cell elongate cylindrical, elongate ellipsoidal, elongate ovoid or ellipsoidal ……………………………………………………………………………… 11

8. Chloroplast cup-shaped …………………………………………… *C. rostafinskii*

8. Chloroplast parietal on the dorsal side of the cell ……………………… 9

9. Chloroplast with a longitudinal slit in the anterior half of the chloroplast; cell aggregates (more than 16 cells) formed in old cultures ………………………… *C. polyptera**

9. Chloroplast without a longitudinal slit in the anterior half of the chloroplast; cell aggregates not formed in old cultures …………………………………………………………… 10

10. Chloroplast not filling in the posterior end of the protoplast; zoospores formed within the parental cell up to eight …………………………………………… *C. fukushimae**

10. Chloroplast filling in the posterior end of the protoplast; zoospores formed within the parental cell up to four …………………………………………… *C. krienitzii**

11. Chloroplast seemingly composed of elongate ovoid or elongate cylindrical platelets ……………………………………………………………………………… 12

11. Chloroplast seemingly composed of angular discs ……………………… 14

12. Cell aggregates formed in old cultures; zoospores formed within the parental cell up to 16 ……………………………………………………………… *C. tughillensis**

12. Cell aggregates not formed in old cultures; zoospores formed within the parental cell up to four or eight ……………………………………………………………………………… 13

13. Cell elongate ellipsoidal; zoospores formed within the parental cell up to eight

……………………………………………………………… *C. hohamii**

13. Cell elongate cylindrical; zoospores formed within the parental cell up to four

……………………………………………………………… *C. tenuis**

14. Eyespot absent …………………………………………………………… 15

14. Eyespot present …………………………………………………………… 17

15. Cell aggregates formed in old cultures ………………………… *C. pichinchae**

15. Cell aggregates not formed in old cultures ………………………………………… 16

16. Maximum cell length more than 20 μm; maximum cell width more than 13 μm ……………………………………………………………… *C. chenangoensis**

16. Maximum cell length less than 20 μm; maximum cell width less than 13 μm ……………………………………………………………… *C. hoshawii* sp. nov.*

17. Cell length less than 15 μm …………………………………………… *C. alpina*

17. Cell length more than 15 μm …………………………………………… *C. remiasii* sp. nov.*

**References**

1. Ettl H. Die gattung *Chloromonas* Gobi emend. Wille (*Chlamydomonas* und die nächstverwandten gattungen I). Nova Hedwigia Beih. 1970;34: 1–283. German.

2. Ettl H. Chlorophyta 1. Phytomonadina. In: Ettl H, Gerloff J, Heynig H, Mollenhauer D, editors. Süßwasserflora von Mitteleuropa 9. Stuttgart: G. Fischer Verlag; 1983. p. 1–807. German.

3. Matsuzaki R, Kawai-Toyooka H, Hara Y, Nozaki H. Revisiting the taxonomic significance of aplanozygote morphologies of two cosmopolitan snow species of the genus *Chloromonas* (Volvocales, Chlorophyceae). Phycologia. 2015;54: 491–502. doi: 10.2216/15-33.1.

4. Ling HU, Seppelt RD. Snow algae of the Windmill Islands, continental Antarctica. 2. *Chloromonas rubroleosa* sp. nov. (Volvocales, Chlorophyta), an alga of red snow. Eur J Phycol. 1993;28: 77–84. doi: 10.1080/09670269300650131.

5. Hoham RW, Bonome TA, Martin CW, Leebens‐Mack JH. A combined 18S rDNA and *rbc*L phylogenetic analysis of *Chloromonas* and *Chlamydomonas* (Chlorophyceae, Volvocales) emphasizing snow and other cold-temperature habitats. J Phycol. 2002;38: 1051–1064. doi: 10.1046/j.1529-8817.2002.t01-1-01227.x.

6. Hoham RW, Berman JD, Rogers HS, Felio JH, Ryba JB, Miller PR. Two new species of green snow algae from Upstate New York, *Chloromonas chenangoensis* sp. nov. and *Chloromonas tughillensis* sp. nov. (Volvocales, Chlorophyceae) and the effects of light on their life cycle development. Phycologia. 2006;45: 319–330. doi: 10.2216/04-103.1.

7. Muramoto K, Nakada T, Shitara T, Hara Y, Nozaki H. Re-examination of the snow algal species *Chloromonas miwae* (Fukushima) Muramoto et al., comb. nov. (Volvocales, Chlorophyceae) from Japan, based on molecular phylogeny and cultured material. Eur J Phycol. 2010;45: 27–37. doi: 10.1080/09670260903272607.

8. Matsuzaki R, Hara Y, Nozaki H. A taxonomic study of snow *Chloromonas* species (Volvocales, Chlorophyceae) based on light and electron microscopy and molecular analysis of cultured material. Phycologia. 2014;53: 293–304. doi: 10.2216/14-3.1.

9. Kirjakov IK, Velichkova KN. New species of green snow algae *Chloromonas* (Volvocales, Chlorophyta) from Bulgaria. International Journal of Fisheries and Aquatic Studies. 2016;4: 94–95.
